# Synthesis and Biological Studies on Dinuclear Gold(I) Complexes with Di-(*N*-Heterocyclic Carbene) Ligands Functionalized with Carbohydrates

**DOI:** 10.3390/molecules25173850

**Published:** 2020-08-24

**Authors:** Federica Tresin, Valentina Stoppa, Marco Baron, Andrea Biffis, Alfonso Annunziata, Luigi D’Elia, Daria Maria Monti, Francesco Ruffo, Marco Roverso, Paolo Sgarbossa, Sara Bogialli, Cristina Tubaro

**Affiliations:** 1 Department of Chemical Sciences, University of Padova, via Marzolo 1, 35131 Padova, Italy; federica.tresin@unipd.it (F.T.); valentina.stoppa@studenti.unipd.it (V.S.); marco.baron@unipd.it (M.B.); andrea.biffis@unipd.it (A.B.); marco.roverso@unipd.it (M.R.); sara.bogialli@unipd.it (S.B.); 2 Department of Chemical Sciences, University of Napoli Federico II, Complesso Universitario di Monte S. Angelo, via Cintia 21, 80126 Napoli, Italy; alfonso.annunziata@unina.it (A.A); luigi.delia@unina.it (L.D.); mdmonti@unina.it (D.M.M.); ruffo@unina.it (F.R.); 3 Department of Industrial Engineering, University of Padova, via Marzolo 9, 35131 Padova, Italy; paolo.sgarbossa@unipd.it

**Keywords:** gold complexes, *N*-heterocyclic carbene, bidentate ligands, oxidative addition, cytotoxicity

## Abstract

The design of novel metal complexes with *N*-heterocyclic carbene (NHC) ligands that display biological activity is an active research field in organometallic chemistry. One of the possible approaches consists of the use of NHC ligands functionalized with a carbohydrate moiety. Two novel Au(I)–Au(I) dinuclear complexes were synthesized; they present a neutral structure with one bridging diNHC ligand, having one or both heterocyclic rings decorated with a carbohydrate functionality. With the symmetric diNHC ligand, the dicationic dinuclear complex bearing two bridging diNHC ligands was also synthesized. The study was completed by analyzing the antiproliferative properties of these complexes, which were compared to the activity displayed by similar mononuclear Au(I) complexes and by the analogous bimetallic Au(I)–Au(I) complex not functionalized with carbohydrates.

## 1. Introduction

Cisplatin was used as an anticancer drug for many years, and it is still one of the most used ones, despite its numerous issues with drug resistance and side effects [1,2]. These issues require more research to find a valid transition metal-based alternative to cisplatin. In this regard, metal complexes with *N*-heterocyclic carbene ligands (NHC) [3,4,5] are attracting increasing attention from the bioinorganic scientific community; these complexes are especially interesting due to their high stability imparted by the strength of the M–NHC bond, such that it appears reasonable that the structure of these complexes remains unchanged and stable under physiological conditions [6,7,8,9,10,11,12]. 

In particular, the interest in gold-based drugs received great impulse from the discovery of the anti-cancer properties of Auranofin, originally used as an antiarthritic drug. Compared to cisplatin, Auranofin presents better activity against difficult to treat tumors, better selectivity, and less cell resistance [13,14]. As Auranofin presents a phosphine ligand, it is reasonable to assume that this ligand could be substituted with an NHC ligand, as NHCs are rapidly substituting phosphine ligands given the higher stability of the resulting complexes; furthermore, as for phosphine ligands, for NHC ones it is also possible to easily and independently modify their steric and electronic properties [15]. This NHC versatility, obtained for example by simply changing the substituents on the nitrogen atoms of the heterocyclic ring, also allows a better fine-tuning of the lipophilic/hydrophilic balance of the molecule, thus enhancing the selectivity of the drug [16]. Many gold(I) and gold(III) complexes with NHC ligands show biological activity, and several reviews appeared on this topic in recent years [10,14,17]. 

The decoration of the carbene ligand with a carbohydrate can be of interest for a variety of reasons; sugars are abundant in nature and present an extremely varied structure, they can enhance the water solubility of the complex, and finally the presence of a sugar residue in the molecule can enhance the drug selectivity as a result of the increased carbohydrate uptake of cancer cells [18,19,20,21,22,23]. The bioactivity of mononuclear gold(I) complexes with carbohydrate-functionalized NHCs were recently studied by some of us [18]. In this manuscript, we report on the synthesis of dinuclear Au(I)–NHC complexes with incorporated acetylated glucopyranose moieties and on their antiproliferative activity. The performances of the dinuclear complexes, in terms of both activity and selectivity, were compared to their corresponding mononuclear counterparts and to dinuclear complexes not having carbohydrate-functionalized NHCs. Dinuclear Au(I) complexes with one or two bridging diNHC carbene ligands were reported to present anticancer properties, acting by inhibiting the thioredoxin reductase TrxR or by leading to mitochondria-induced apoptosis [24,25,26,27,28,29,30,31]. 

## 2. Results and Discussion

### 2.1. Synthesis of the Bis(Imidazolium) Salts

**Scheme 1:**
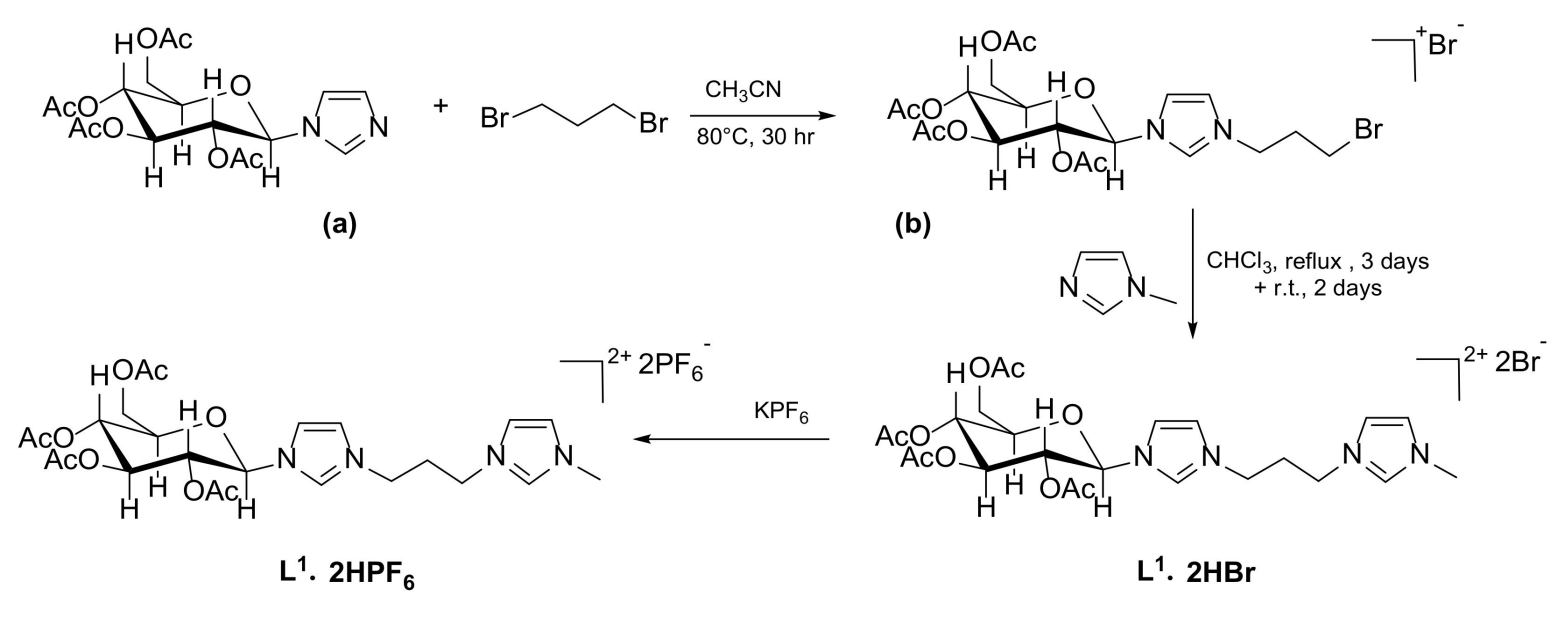
Synthesis of the bis(imidazolium) salt **L^1^∙2HPF_6_**; (**a**) carbohydrate-functionalized imidazole; (**b**) imidazolium salt.

Compound **L^1^∙2HPF_6_** was synthesized following a three-step process (Scheme 1), in which the carbohydrate-functionalized imidazole (**a**) [32] reacts with 1,3-dibromopropane to obtain the imidazolium salt (**b**). This reaction does not yield the bis(imidazolium) symmetric product, possibly due either to the limited nucleophilicity of the imidazole (**a**) or to the low reactivity of dibromopropane under the reaction conditions. In fact, by reacting product (**b**) with *N*-methylimidazole, compound **L^1^∙2HBr** can be isolated. The final Br^−^/PF_6_^−^ anion metathesis step is usually required to isolate an azolium salt more soluble in organic solvents, such as acetonitrile, and to prevent interferences of the counter anions during the synthesis of the Au(I) complexes, especially the dinuclear dicationic ones.

**Scheme 2:**
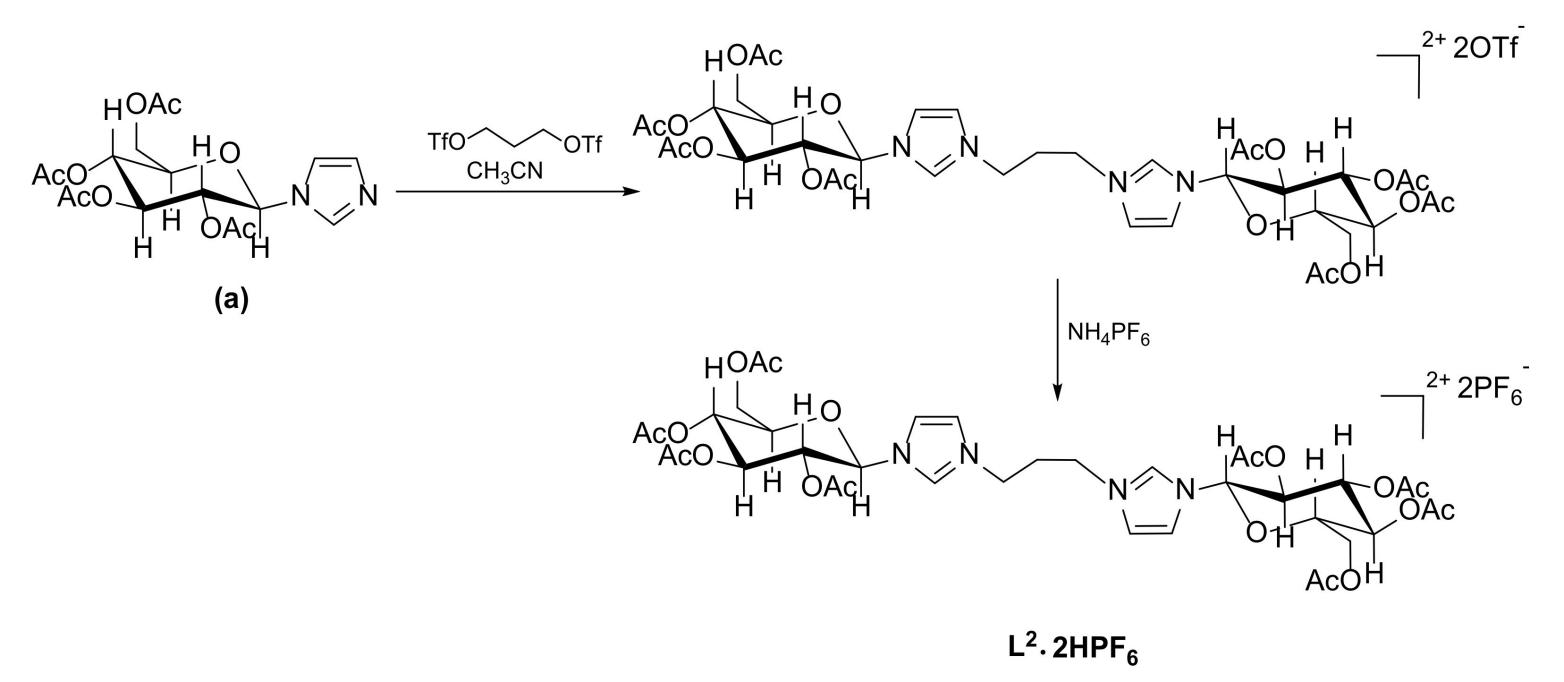
Synthesis of the bis(imidazolium) salt **L^2^∙2HPF_6_**; (**a**) carbohydrate-functionalized imidazole.

In order to obtain the symmetric bis(imidazolium) salt **L^2^∙2HPF_6_**, imidazole (**a**) was reacted with 1,3-propylenebistriflate, a substrate more activated than 1,3-dibromopropane for the nucleophilic substitution, following a procedure reported by Anneser et al. (Scheme 2) [33]. Also in this case, the final step is the anion metathesis. 

Both **L^1^∙2HPF_6_** and **L^2^∙2HPF_6_** were characterized by ^1^H- and ^13^C{^1^H}-NMR spectroscopy, as well as electrospray ionization mass spectrometry (ESI-MS), and both salts appear to be spectroscopically pure. In the case of compound **L^1^∙2HPF_6_**, the acidic protons of the imidazole C2-H hydrogens give two signals in the ^1^H-NMR spectrum at 8.72 and 8.73 ppm, indicating the lack of symmetry of the system. Conversely, the ^1^H-NMR spectrum of symmetric **L^2^∙2HPF_6_** presents only one peak at 8.82 ppm. Anomerization processes were reported in the literature during the quaternization of the imidazole ring or during the synthesis of carbene complexes with carbohydrate functionalized NHC [34]. In both compounds, the signal relative to the anomeric proton, which is found around 5.7–5.8 ppm, has a coupling constant value of around 9 Hz. Comparing this *^3^J_HH_*value with data found in literature relative to the anomeric proton in glucopyranose rings, it is reasonable to state that the carbohydrate remains present in the β anomer form in both diazolium salts; the coupling constant of the same peak for the α anomer is, in fact, much lower, around 2–4 Hz [35]. 

### 2.2. Synthesis of the [Au_2_Br_2_L] Complexes

**Scheme 3:**
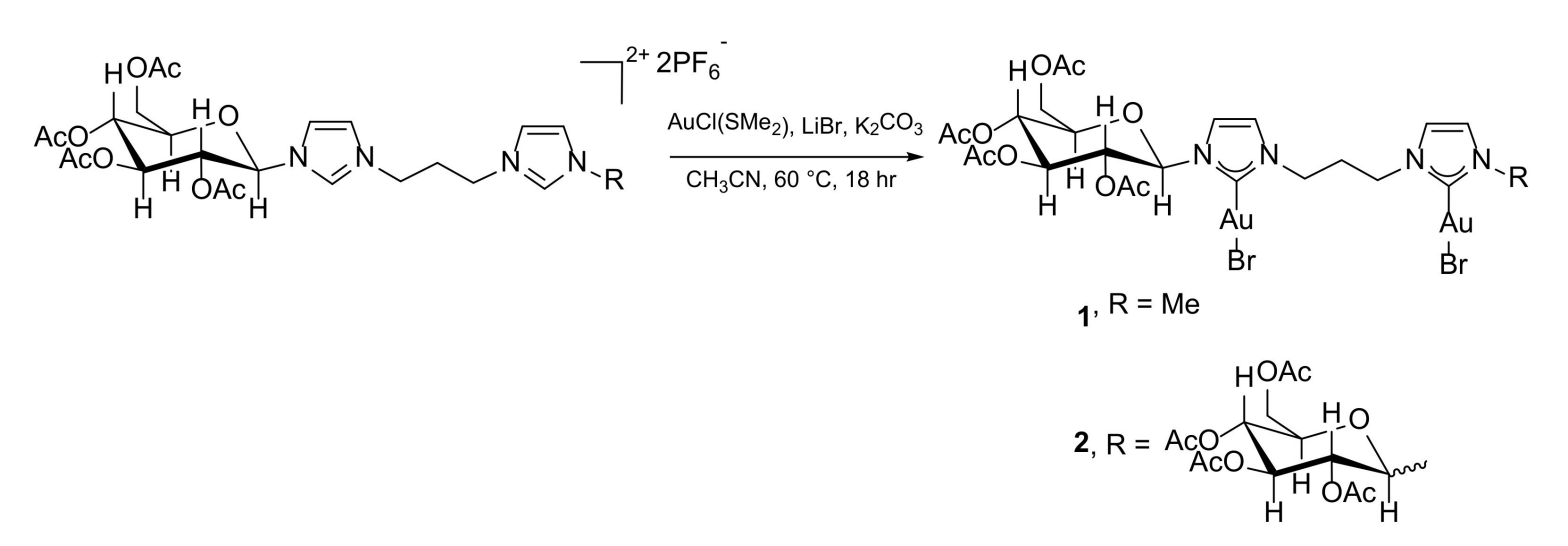
Synthesis of the dinuclear gold(I) complexes **1** and **2**.

Compounds **1** and **2** were synthesized following a single-step procedure already reported in the literature [36] in which the proper bis(imidazolium) salt reacts with the gold precursor AuCl(SMe_2_) in the presence of LiBr and K_2_CO_3_ as a base to deprotonate the bis(imidazolium) salt. The addition of LiBr prevents the formation of the analogous chloro complexes [Au_2_Cl_2_L]; furthermore, it was reported by Nolan and co-workers that the anion which usually coordinates to gold(I) in the complexes is that of the starting azolium salt [37]. The neutral [Au_2_Br_2_L] complexes (Scheme 3) were then characterized by ^1^H- and ^13^C{^1^H}-NMR spectroscopy, as well as ESI-MS. In particular, an indication that the complexes formed is provided by the disappearance of the peak relative to the acidic C2-Hs, supporting the deprotonation of the diazolium salt. Further proof comes from the ^13^C-NMR spectrum, in which the peak relative to the imidazole C2 is present at around 170–175 ppm, in the range of values found in the literature for carbene carbons coordinated to a gold(I) center *trans* to a bromide ligand [36,38]. In both complexes, the carbohydrate is present in the β anomeric form as suggested by the value (9 Hz) of the *^3^J_HH_* coupling constant of the anomeric proton in the glucopyranose ring [35]. From the ESI-MS spectra, the most prominent peaks are the [Au_2_BrL]^+^ (at *m*/*z* 993 and 1311 for compounds **1** and **2**, respectively) and the [Au_2_Br_2_LK]^+^ cations (*m*/*z* 1113 and 1429 for compounds **1** and **2**, respectively).

### 2.3. Synthesis of the [Au_2_L_2_](PF_6_)_2_ Complexes

**Scheme 4:**
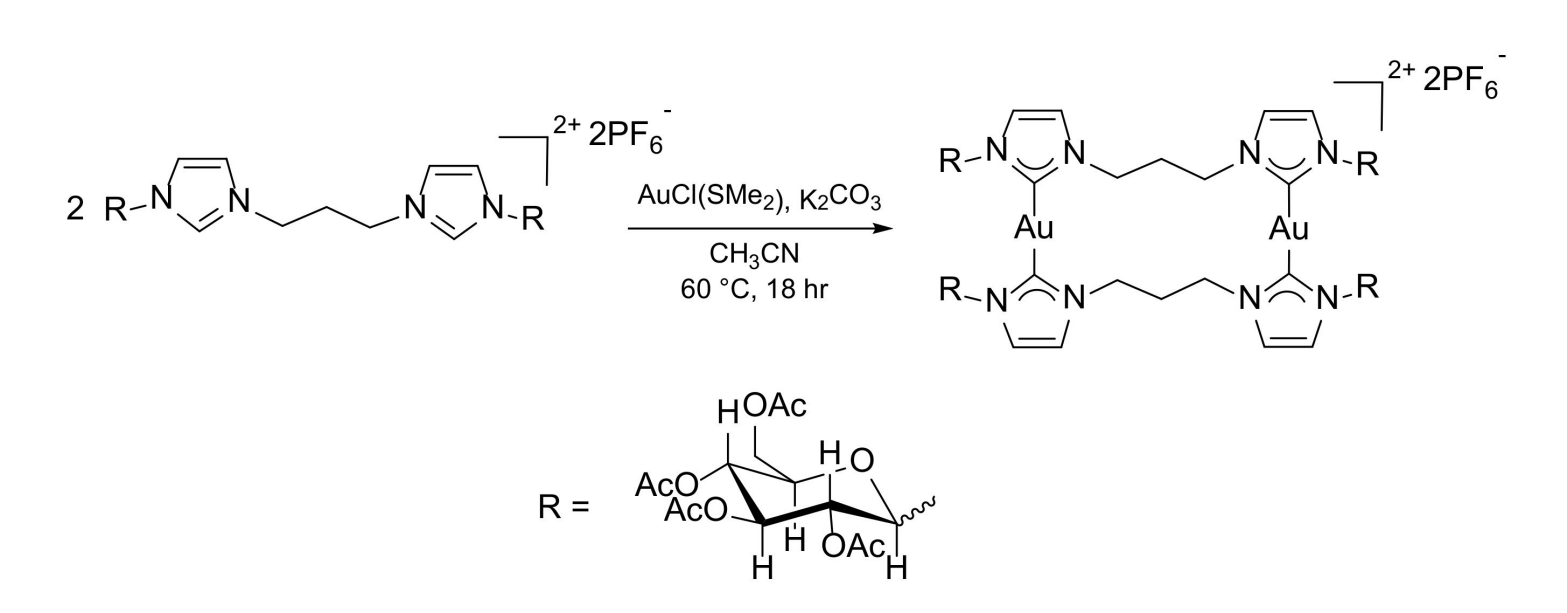
Synthesis of the dinuclear gold(I) complex **3**.

With this type of dicarbene ligand, in addition to the neutral complexes described in the previous section, it is possible to also isolate dinuclear dicationic complexes with the general formula [Au_2_L_2_]^2+^ having two ligands bridging the two gold centers. Considering the asymmetric nature of the proligand **L^1^∙2HPF_6_**, at least two configurational isomers can be isolated: one with the carbohydrate-imidazoles facing each other and one with each carbohydrate-NHC facing a methylimidazole-2-ylidene. Furthermore, we recently reported that, in similar [Au_2_L_2_]^2+^ complexes with heteroditopic ligands, the number of possible products is also increased by the different conformations of the propylene linkers between the carbene units [39,40]. For this reason, we investigated only the reaction with the symmetric ligand L^2^. Compound **3** was synthesized following the same procedure described for complex **2** but using a 1:1 **L^2^∙2HPF_6_**:AuCl(SMe_2_) molar ratio and without adding LiBr to the reaction mixture (Scheme 4). Once again, the formation of the complex is supported by the disappearance in the ^1^H-NMR spectrum of the peak associated to the C2-H on the imidazole rings. The stoichiometry of the complex was confirmed by high-resolution mass spectrometry measurements where a peak relative to the dicationic [Au_2_L^2^_2_]^2+^ fragment is present at 1033.2594 *m*/*z*. Another indication that this complex is cationic comes from the ^13^C{^1^H}-NMR spectrum; the peak of the carbene carbon is found at 183.2 ppm, downfield shifted by ca. 10 ppm with respect to the signal observed in the corresponding neutral complex **2** (174.6 ppm). This chemical shift value is coherent with the values reported in literature for carbene carbons with another carbene carbon in *trans* position [41,42,43,44,45], and this geometry is frequently observed in cationic gold(I) complexes with two carbene ligands coordinated to the same metal center.

### 2.4. Reactivity of the Gold(I) Complex **3** toward Oxidative Addition of Halogens

**Scheme 5:**
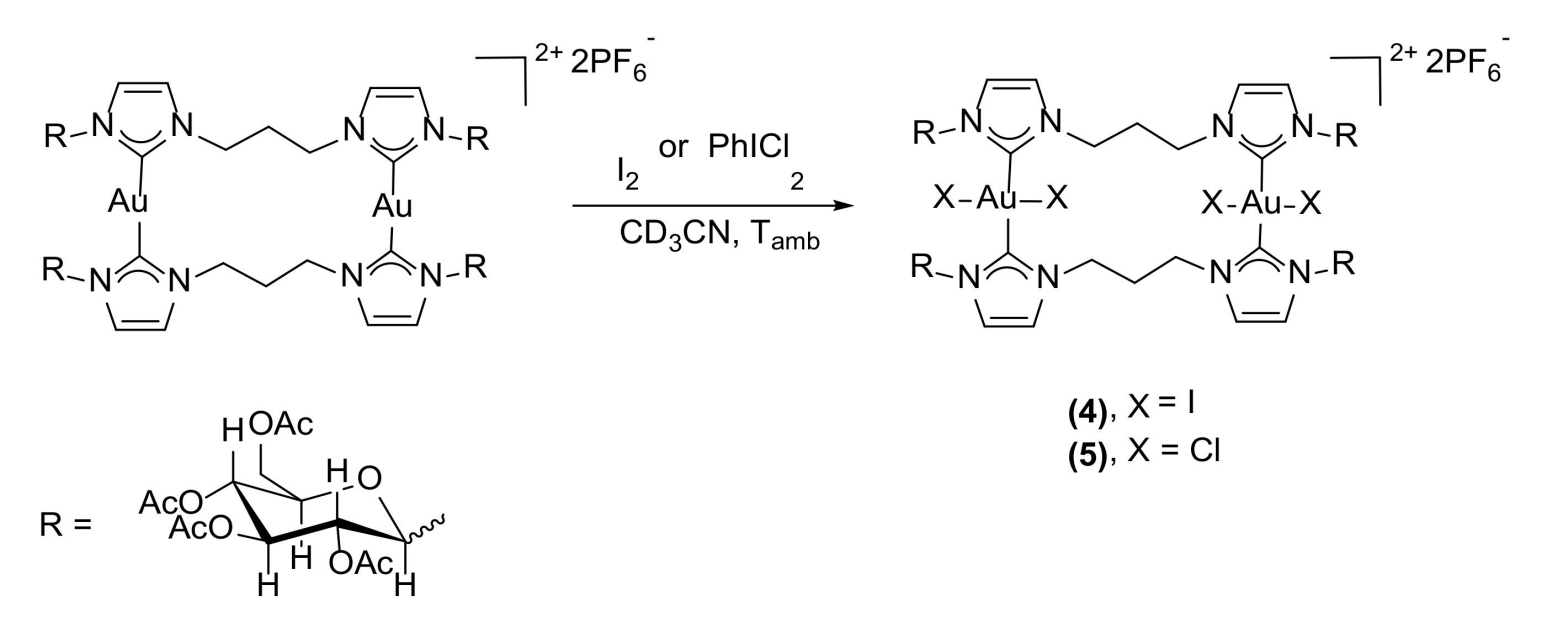
Synthesis of the dinuclear Au(III) complexes by oxidative addition to **3**.

**Figure 1:**
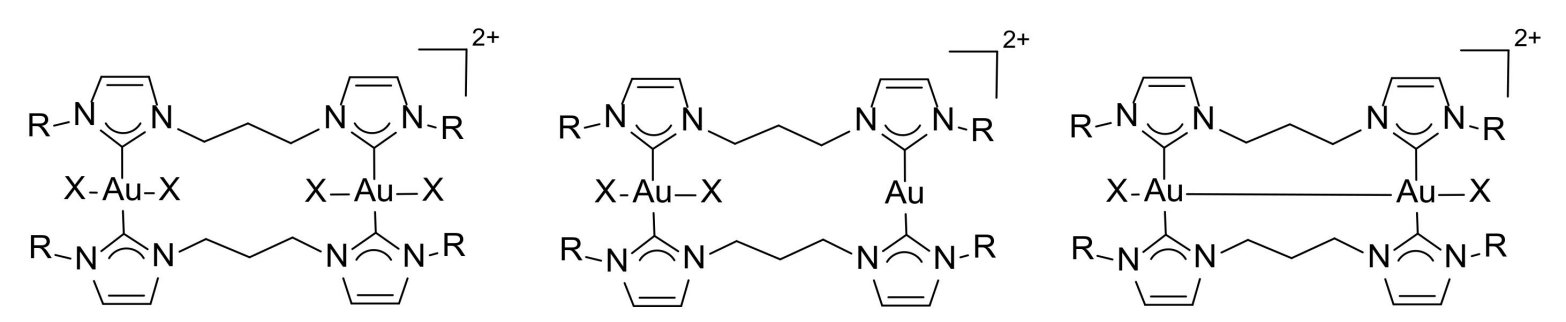
Possible products obtained from the oxidative addition of halogens to the dinuclear gold(I)–gold(I) complexes.

The reactivity of the gold(I) complex **3** in the oxidative addition of halogens to gold was investigated; the reactions were performed in an NMR tube at room temperature in deuterated acetonitrile as solvent, using a slight excess of oxidant (I_2_:[Au] = 1.2:1 and PhICl_2_:[Au] = 1.5:1) (Scheme 5). Many possible products can be obtained from the oxidative addition of halogens to dinuclear diNHC gold(I) complexes: the fully oxidized gold(III)–gold(III) product, the mixed-valence gold(I)–gold(III) complex, and the gold(II)–gold(II) species (Figure 1) [46]. Therefore, the reactions were followed by recording ^1^H-NMR spectra before the addition of the oxidant, immediately after, and three hours and 24 h later in order to monitor any possible changes in the product distribution. With both halogens, the dinuclear gold(III) complex was immediately formed, and no further evolution of the product was detected. 

In both cases, the symmetry of the complex is maintained, as shown by the ^1^H-NMR spectra which present only one set of peaks relative to the sugars and imidazole rings. This suggests that the two metal centers are equivalent and, therefore, present the same oxidation state. The ^13^C-NMR spectra show more definite proof that the oxidation state of the gold centers changed from gold(I) to gold(III); the carbene carbons present, in fact, a peak at 145.6 ppm for complex **4** and 154.6 ppm for **5**. These values of chemical shifts are 20–30 ppm lower than the value (δ 183.2 ppm) found for complex **3**. This is usually explained taking into consideration the more pronounced Lewis acidic behavior of gold(III), causing an extended delocalization of the π electron density of the imidazole C=C double bond toward the carbene carbon [47,48]. The difference between the ^13^C-NMR carbene chemical shifts in **4** and **5** is ca. 10 ppm and is due to the different nature of the two halide ligands [46]. The definitive proof that both complexes are dinuclear gold(III) complexes comes from the high-resolution mass spectra; for both complexes, the most prominent signal is the one given by the [Au_2_X_4_L_2_]^2+^ ion, at *m*/*z* 1287.0697 for **4** and 1103.1974 for **5**, both for monoisotopic peaks.

### 2.5. Biological Activity of the Gold(I) Complexes

**Figure 2:**
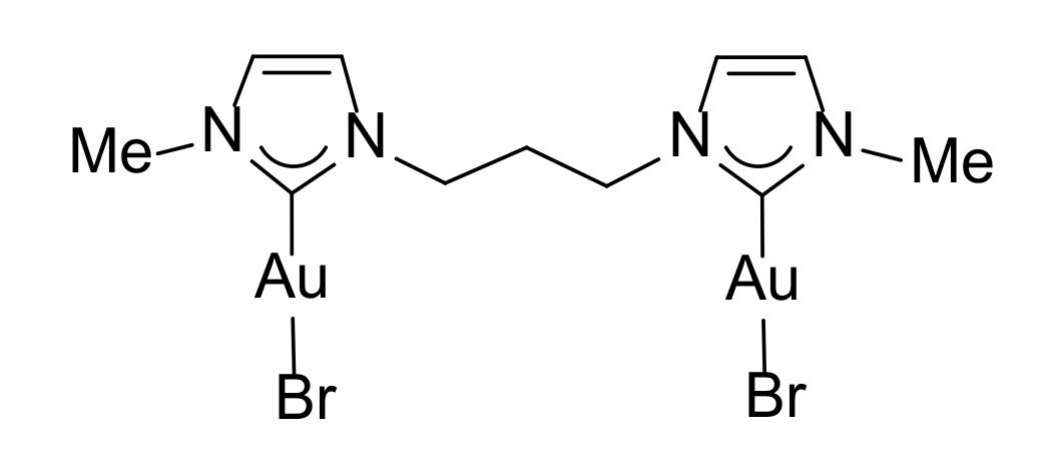
Structure of complex **6**.

The biological activity of the gold(I) complexes **1**–**3** and **6** was tested on different eukaryotic cell lines. Complex **6** (Figure 2), bearing only methyl groups as wingtip substituents, was chosen for the absence of any carbohydrate moiety as comparison.

**Figure 3:**
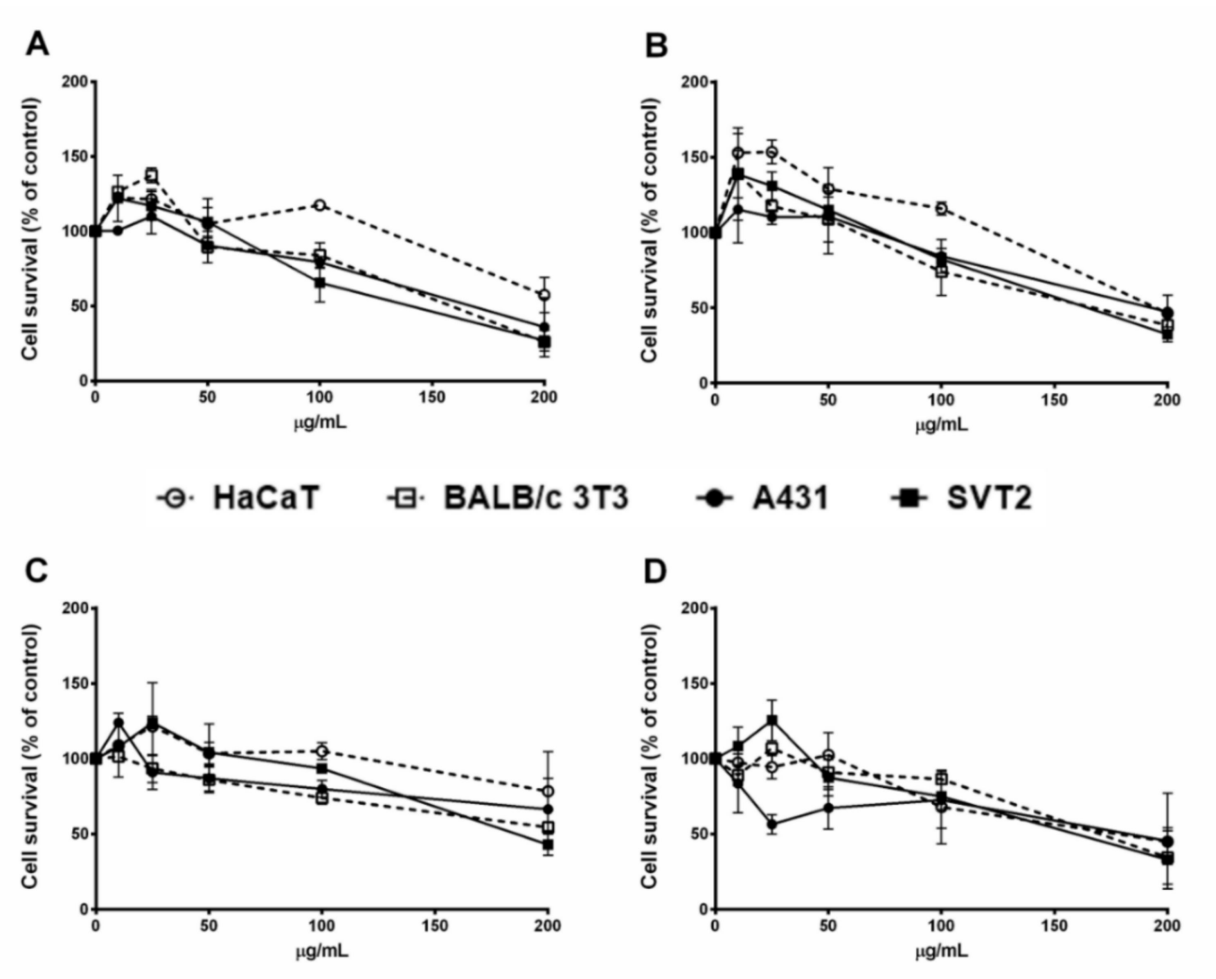
Effect of complexes **1**, **2**, **3**, and **6** on the survival of different cell lines. Immortalized human cells (HaCaT, dashed line with empty circles), immortalized murine cells (BALB/c-3T3, dashed line with empty squares), human epidermoid carcinoma (A431, black line with black circles), and murine fibroblast transformed with simian virus 40 (SV40) (SVT2, black line with black squares) were incubated with increasing amounts of each compound (10–200 µg/mL) for 48 h. Cell viability was assessed by 3-(4,5-dimethylthiazol-2-yl)-2,5-diphenyltetrazolium bromide (MTT) assay and expressed as described in the Section 3. **A**, complex **1**; **B**, complex **2**; **C**, complex **3**; **D**, complex **6**. Values are given as means ± SD (*n* ≥ 3).

The assay was performed to study the importance of the functionalization with a carbohydrate moiety in determining the antiproliferative activity. We did not test the antiproliferative activity of gold(III) complexes **4** and **5**, as it is well known that similar dicationic gold(III) complexes easily undergo reduction to the corresponding gold(I) species in a physiological environment [26,48]. In particular, two cancer cell lines, A431 and SVT2, were tested in the presence of increasing amount of each compound. Immortalized cell lines, HaCaT and BALB/c-3T3, were analyzed as well to study the selectivity of the newly synthesized compounds. Cell viability was evaluated by the 3-(4,5-dimethylthiazol-2-yl)-2,5-diphenyltetrazolium bromide (MTT) assay and the results, after 48 h of incubation, are reported in Figure 3. All the complexes were cytotoxic on all cell lines analyzed, and they showed a dose-dependent toxicity. Interestingly, the analyzed compounds induced an increase in cell proliferation at very low concentration (5–10 µg/mL). The IC_50_ values, i.e., the complex concentration required to induce 50% of cell death, is reported in Table 1. Complex **3** was the compound with the lowest toxicity with respect to the other tested molecules. 

Even if the IC_50_ values for the reported dinuclear complexes are very high (>100 μM), these results are not totally unexpected, as this inertness was also observed for the mononuclear complex [Au(magi)Cl] (magi = 1-methyl-3-(2,3,4,6-tetra-*O*-acetyl-β-d-glucopyranosyl)imidazole-2-ylidene) [18] and was attributed to the low mitochondrial penetration of the complex. Comparing the performances of complexes **1**, **2**, and **6**, it is evident that the introduction of a sugar in the carbene moiety only slightly improves the cytotoxic activity. Complex **3** shows lower IC_50_ values than those of the neutral complex **2**, having the same diNHC ligand. 

In general, halo-substituted neutral gold(I) complexes turn out to be less effective than their corresponding cationic bis(NHC) complexes. The lability of the Au–X (X = halogen) bond, compared to the relative inertness of the Au–NHC bonds, makes the halide derivatives less stable in biologically relevant conditions, favoring the occurrence of deactivation reactions by different cellular components. Another parameter that can explain the lower activity of the neutral gold(I) complexes is their lower solubility in water, which possibly reduces the drug uptake by cells [49]. Finally, the higher activity of complex **3** can be also attributed to its dicationic nature, which allows its classification as a delocalized lipophilic cation (DLC) [50]. Indeed, the difference in the mitochondrial membrane potential between cancerous and healthy cells could explain the higher penetration of DLCs into the mitochondrial membrane of tumor cells, which in turn would lead to cell apoptosis [51].

## 3. Materials and Methods 

### 3.1. General Comments

All commercially available reagents (Sigma-Aldrich, Darmstadt, Germany) were used as received without additional purification steps. The reagents **a** [32], **b** [52], 1,3-propylenebistriflate [53], and complex **6** [36] were prepared according to literature procedures. The NMR spectra were recorded on a Bruker Avance 300 (Bruker, Billerica, MA, USA; 300.1 MHz for ^1^H and 75.5 MHz for ^13^C) at 298 K unless otherwise stated; chemical shifts (δ) are reported in units of ppm relative to the residual solvent signals. ESI-MS analyses of compounds **L^1^∙2HPF_6_**, **L^1^∙2HPF_6_**, **1**, and **2** were performed using an LCQ-Duo (Thermo Fisher Scientific, Waltham, Massachusetts, USA) operating in positive ion mode; sample solutions were prepared by dissolving the compounds in acetonitrile and were directly infused into the ESI source by a syringe pump at 8 μL/min flow rate. The HRMS measures of complexes **3**–**5** were performed using a Q-Exactive hybrid quadrupole-Orbitrap™ mass spectrometer (Thermo Fisher Scientific). MS conditions were as follows: electrospray ionization in positive mode, resolution 70,000, automatic gain control (AGC) target 1 × 10^6^, max injection time of 50 ms, scan range 500–2000 amu, capillary voltage 3.5 kV and radiofrequency (RF) voltage 50 V, capillary temperature 320 °C and probe temperature 350 °C; nitrogen was used as sheath gas at 11 psi. Samples were prepared using acetonitrile as solvent and injected for analysis at a flow rate of 10 μL/min. Calibration was performed with a standard solution purchased from Thermo Fisher Scientific (Pierce® ESI positive Ion Calibration Solution). The software for analysis of MS data was Xcalibur 3.1 (Thermo Fisher Scientific). Elemental analyses were carried out by the microanalytical laboratory of Chemical Sciences Department (University of Padova) with a Thermo Scientific FLASH 2000 apparatus. The recorded NMR and ESI-MS spectra of the reported compounds can be found in the Supplementary Materials section.

**Figure 4:**
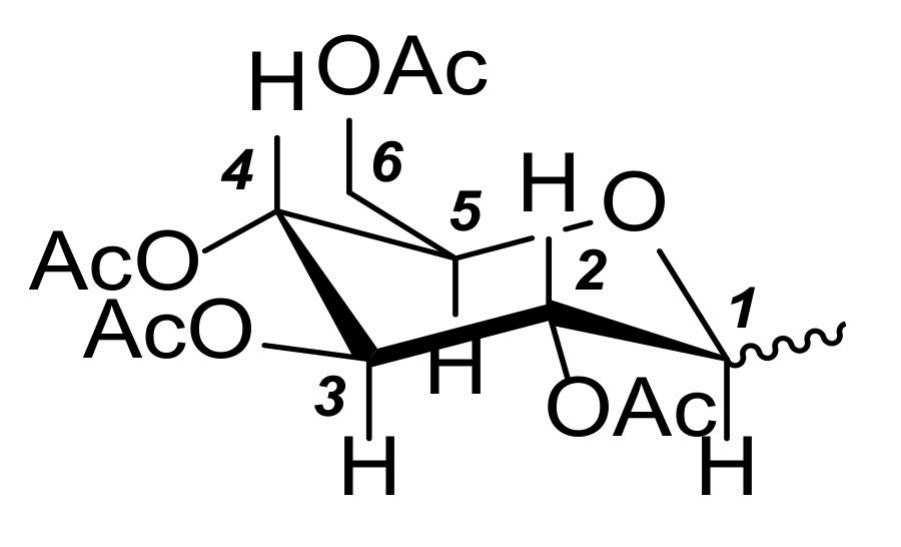
Numbering of the carbon atoms in the sugar substituent for NMR assignments.

In the characterization of the imidazolium salts and gold complexes, the following notation (Figure 4) was adopted for the sugar substituent:

### 3.2. Synthesis of the Bis(Imidazolium) Salts

#### 3.2.1. Synthesis of the Bis(Imidazolium) Salt **L^1^∙2HPF_6_**

Compound **b** (0.19 g, 0.32 mmol), *N*-methylimidazole (91 μL, 1.14 mmol), and 10 mL of CHCl_3_ were added to an Ace pressure tube. The solution was stirred at 65 °C for three days, then for two additional days at room temperature. The solvent was evaporated under reduced pressure to give a white solid. The solid product was dissolved in 5 mL of a saturated aqueous KPF_6_ solution and left stirring overnight. The formation of an oil was observed. The oily residue was separated from the aqueous solution and dried under vacuum. The residue was treated with 20 mL of diethyl ether under stirring for three hours and the formation of a white solid was observed. The solid was isolated by filtration (yield 44%). ^1^H-NMR (300 MHz, CD_3_CN) δ 8.76 (s, 1H, NCHN-GluIm), 8.41 (s, 1H, NCHN-MeIm), 7.64 (s, 1H, GluIm), 7.47 (s, 1H, GluIm), 7.38 (m, 2H, MeIm), 5.74 (d, *^3^J* = 9.0 Hz, 1H, 1-Glu), 5.50 (m, 1H, 3-Glu), 5.38–5.21 (m, 2H, 2,4-Glu), 4.32–4.18 (m, 3H, 5,6-Glu), 4.18–4.09 (m, 4H, NCH_2_), 3.84 (s, 3H, CH_3_-MeIm), 2.38 (quint, 2H, *^3^J* = 7.2 Hz, 2H, CH_2_), 2.07 (s, 3H, CH_3_-Ac), 2.03 (s, 3H, CH_3_-Ac), 1.99 (s, 3H, CH_3_-Ac), 1.94 (s, 3H, CH_3_-Ac). ^13^C{^1^H}-NMR (75 MHz, CD_3_CN) δ 171.16 (CO), 170.59 (CO), 170.48 (CO), 170.43 (CO), 136.91 (NCHN-GluIm), 136.31 (NCHN-MeIm), 124.78 (GluIm), 124.12 (GluIm), 123.01 (MeIm), 121.61 (MeIm), 85.28 (1-Glu), 75.56 (Glu), 72.13 (Glu), 71.87 (Glu), 67.97 (Glu), 62.08 (6-Glu), 47.64 (NCH_2_), 46.76 (NCH_2_), 36.67 (CH_3_-MeIm), 30.46 (CH_2_), 20.63 (CH_3_-Ac), 20.56 (CH_3_-Ac), 20.33 (CH_3_-Ac). ESI-MS (positive ions, CH_3_CN): *m*/*z* 667 [H_2_L^1^PF_6_]^+^, 983 [H_2_L^2^PF_6_]^+^, 1479 [(H_2_L^1^)_2_(PF_6_)_3_]^+^. Elemental analysis C_24_H_34_N_4_O_9_P_2_F_12_. Calculated: C, 35.48%; H, 4.22%; N, 6.90%. Found: C, 34.96%; H, 3.73%; N 5.57%. 

#### 3.2.2. Synthesis of the Bis(Imidazolium) Salt **L^2^∙2HPF_6_**

Compound **a** (231 mg, 0.58 mmol) was dissolved in 50 mL of acetonitrile at 0 °C. A solution of 1,3-propylenebistriflate (98 mg, 0.29 mmol) in 7 mL of acetonitrile was prepared and added dropwise, over one hour, to the solution containing **a**. The resulting solution was warmed to room temperature and left stirring for 16 h. The solvent was removed and the obtained solid was dissolved in 5 mL of distilled water. A saturated aqueous NH_4_PF_6_ solution (5 mL) was added, and the final solution was left stirring for an hour until the formation of a white solid precipitate was observed. The solid was filtered, washed with distilled water, and then dried under reduced pressure (yield 48%). ^1^H-NMR (300 MHz, CD_3_CN) δ 8.82 (s, 2H, NCHN-Im), 7.67 (t, *^3^J* = 1.9 Hz, 2H, Im), 7.50 (t, *^3^J* = 1.9 Hz, 2H, Im), 5.78 (d, *^3^J* = 9.0 Hz, 2H, 1-Glu), 5.52 (m, 2H, 3-Glu), 5.38–5.25 (m, 4H, 2,4-Glu), 4.28–4.22 (m, 6H, 5,6-Glu), 4.22–4.17 (m, 4H, NCH_2_), 2.47–2.36 (m, 2H, CH_2_), 2.05 (s, 6H, CH_3_-Ac), 2.05 (s, 6H, CH_3_-Ac), 2.01 (s, 6H, CH_3_-Ac). ^13^C{^1^H}-NMR (75 MHz, CD_3_CN) δ 171.2 (CO), 170.6 (CO), 170.5 (CO), 136.5 (NCHN-Im), 124.2 (Im), 121.8 (Im), 85.4 (1-Glu), 75.7 (3-Glu), 72.2 (Glu), 71.9 (Glu), 68.0 (Glu), 62.1 (Glu), 47.6 (NCH_2_), 30.4 (CH_2_), 20.7 (CH_3_-Ac), 20.7 (CH_3_-Ac), 20.5 (CH_3_-Ac). ESI-MS (positive ions, CH_3_CN): *m*/*z* 983 [H_2_L^2^PF_6_]^+^, 507 [H_2_L^2^-Glu]^+^, 419 [H_2_L^2^]^2+^. Elemental analysis C_37_H_50_N_4_O_18_P_2_F_12_. Calculated: C, 39.37%; H, 4.46%; N, 4.96%. Found: C, 39.07%; H, 4.24%; N, 4.36%.

### 3.3. General Procedure for the Synthesis of Complexes [Au_2_Br_2_L]

The proper bis(imidazolium) salt (0.086 mmol), AuCl(SMe_2_) (0.172 mmol), K_2_CO_3_ (1.89 mmol), LiBr (0.86 mmol), and 40 mL of acetonitrile were added to a round-bottom flask. The mixture was heated to 60 °C and left stirring for 18 h, then filtered on Celite to remove excess salts. The solvent was removed under reduced pressure. The solid was finally recrystallized with chloroform/*n*-hexane (for **1**) or acetonitrile/diethyl ether (for **2**), obtaining a white solid product which was filtered and dried under vacuum. 

**1.** White solid, yield 59%. ^1^H-NMR (300 MHz, CD_3_CN) δ 7.43 (d, *^3^J* = 2.1 Hz, 1H, GluIm), 7.29 (d, *^3^J* = 2.1 Hz, 1H, GluIm), 7.20 (d,*^3^J* = 1.9 Hz, 1H, MeIm), 7.17 (d, *^3^J* = 1.9 Hz, 1H, MeIm), 6.12 (d,*^3^J* = 8.9 Hz, 1H, 1-Glu), 5.52 (m, 1H, 3-Glu), 5.33–5.19 (m, 2H, 2,4-Glu), 4.27–4.17 (m, 3H, 5,6-Glu), 4.17–4.02 (m, 4H, NCH_2_), 3.85 (s, 3H, CH_3_-MeIm), 2.50–2.35 (m, 2H,CH_2_), 2.06 (s, 3H, CH_3_-Ac), 2.04 (s, 3H, CH_3_-Ac), 1.98 (s, 3H, CH_3_-Ac), 1.93 (s, 3H, CH_3_-Ac). ^13^C{^1^H}-NMR (75 MHz, CD_3_CN) δ 175.07 (NCN-MeIm), 172.91 (NCN-GluIm), 171.27 (CO), 170.66 (CO), 170.58 (CO), 169.99 (CO), 123.93 (MeIm), 122.30 (GluIm), 120.73 (MeIm), 119.62 (GluIm), 86.64 (1-Glu), 75.13 (3-Glu), 72.64 (Glu), 72.24 (Glu), 68.51 (Glu), 62.34 (Glu), 48.31 (NCH_2_), 47.50 (NCH_2_), 38.75 (CH_3_-MeIm), 30.96 (CH_2_), 21.08 (CH_3_-Ac), 20.95 (CH_3_-Ac), 20.82 (CH_3_-Ac), 20.75 (CH_3_-Ac). ESI-MS (positive ions, CH_3_CN): *m*/*z* 993 [Au_2_L^1^Br]^+^, 1113 [Au_2_L^1^Br_2_K]^+^. 

**2**. White solid, yield 89%. ^1^H-NMR (300 MHz, CD_3_CN) δ 7.45 (d, *^3^J* = 2.0 Hz, 2H, Im), 7.30 (d, *^3^J* = 2.0 Hz, 2H, Im), 6.14 (d, *^3^J* = 8.9 Hz, 2H, 1-Glu), 5.54 (m, 2H, 3-Glu), 5.26 (m, 4H, 2,4-Glu), 4.34–4.18 (m, 6H, 5,6-Glu), 4.18–3.98 (m, 4H, NCH_2_), 2.49–2.35 (m, 2H, CH_2_), 2.06 (s, 6H, CH_3_-Ac), 2.04 (s, 6H, CH_3_-Ac), 1.98 (s, 6H, CH_3_-Ac), 1.92 (s, 6H, CH^3^-Ac). ^13^C{^1^H}-NMR (75 MHz, CD_3_CN) δ 174.64 (NCN-Im), 171.24 (CO), 170.65 (CO), 170.59 (CO), 170.01 (CO), 122.34 (Im), 119.67 (Im), 86.76 (1-Glu), 75.20 (Glu), 72.65 (Glu), 72.33 (Glu), 68.55 (Glu), 62.36 (6-Glu), 48.22 (NCH_2_), 21.05 (CH_3_-Ac), 20.97 (CH_3_-Ac), 20.85 (CH_3_-Ac), 20.78 (CH_3_-Ac). ESI-MS (positive ions, CH_3_CN): *m*/*z* 1311 [Au_2_L^2^Br]^+^, 1429 [Au_2_L^2^Br_2_K]^+^. 

### 3.4. Synthesis of the Complex [Au_2_L^2^_2_](PF_6_)_2_, **3**

The salt **L^2^∙2HPF_6_** (76 mg, 0.067 mmol), AuCl(SMe_2_) (20 mg, 0.067 mmol), K_2_CO_3_ (203 mg, 1.47 mmol), and 40 mL of acetonitrile were added to a round-bottom flask; the mixture was heated to 60 °C and left stirring for 18 h, then filtered on Celite to remove excess salts. The solvent was removed at reduced pressure. The residue was recrystallized with acetonitrile/diethyl ether, obtaining a white solid which was filtered and dried under vacuum (yield 69%). ^1^H-NMR (300 MHz, CD_3_CN) δ 7.58 (d, *^3^J* = 1.9 Hz, 4H, Im), 7.42 (d, *^3^J* = 1.9 Hz, 4H, Im), 6.08 (d, *^3^J* = 8.9 Hz, 4H, 1-Glu), 5.63 (m 4H, 3-Glu), 5.35 (m, 8H, 2,4-Glu), 4.47–4.31 (m, 8H, NCH_2_), 4.31–4.13 (m, 12H, 5,6-Glu), 2.54–2.38 (m, 4H,CH_2_), 2.05 (s, 12H, CH_3_-Ac), 2.01 (s, 12H, CH_3_-Ac), 2.01 (s, 12H, CH_3_-Ac), 1.92 (s, 12H, CH_3_-Ac). ^13^C{^1^H}-NMR (75 MHz, CD_3_CN) δ 183.17 (Im), 171.16 (CO), 170.73 (CO), 170.55 (CO), 170.09 (CO), 126.84 (Im), 123.80 (Im), 87.14 (1-Glu), 75.51 (Glu), 72.70 (Glu), 72.58 (Glu), 68.29 (Glu), 62.26 (Glu), 50.11 (NCH_2_), 32.53 (CH_2_), 21.04 (CH_3_-Ac), 20.83 (CH_3_-Ac), 20.80 (CH_3_-Ac). HRMS (positive ions, monoisotopic peak): *m*/*z* 991.2506 [Au_2_L^2^_2_(-2CH_2_CO)]^2+^ (calculated for C_70_H_92_Au_2_N_8_O_34_^2+^ = 991.2518), 1012.2554 [Au_2_L^2^_2_(-CH_2_CO)]^2+^ (calculated for C_72_H_94_Au_2_N_8_O_35_^2+^ = 1012.2571), 1033.2594 [Au_2_L^2^_2_]^2+^ (calculated for C_74_H_96_Au_2_N_8_O_36_^2+^ = 1033.2624).

### 3.5. Reactivity of Complex **3** in Halogen Oxidative Addition: Characterization of the Dinuclear Au(III) Complexes [Au_2_L^2^_2_X_4_](PF_6_)_2_, **4** and **5**

Complex **3** was added to an NMR tube and dissolved in CD_3_CN; the oxidant (I_2_ or PhICl_2_) was then added in a [I_2_]/[Au] = 1.2 and [Cl_2_]/[Au] = 1.5 ratio. NMR spectra were recorded before the addition of the oxidant, immediately after, and three hours and 24 hours later to monitor the reaction. In the case of the [Au_2_L^2^_2_Cl_4_](PF_6_)_2_ complex, the product was isolated as a white solid by removal of the deuterated solvent and by treatment of the residue with diethyl ether to remove the coproduct of the oxidant (PhI). These reactivity tests were run on NMR scale and, for this reason, the characterization of the complexes involved only NMR and MS analysis.

**4**. ^1^H-NMR (300 MHz, CD_3_CN) δ 7.77 (d, *^3^J* = 2.1 Hz, 4H, Im), 7.62 (d,*^3^J* = 2.1 Hz, 4H, Im), 5.90 (d, *^3^J* = 9.3 Hz, 4H, 1-Glu), 5.66 (m, 4H, 3-Glu), 5.40 (m, 4H, 2-Glu), 5.24 (m, 4H, 4-Glu), 4.28–4.00 (m, 20H, 5,6-Glu and NCH_2_), 2.66–2.43 (m, 4H, CH_2_), 1.96 (s, 12H, CH_3_-Ac), 1.94 (s, 12H, CH_3_-Ac), 1.89 (s, 12H, CH_3_-Ac). ^13^C{^1^H}-NMR (75 MHz, CD_3_CN) δ 171.06 (CO), 171.01 (CO), 170.66 (CO), 170.64 (CO), 145.63 (NCN-Im), 126.58 (Im), 123.81 (Im), 85.81 (1-Glu), 75.96 (Glu), 72.69 (Glu), 72.29 (Glu), 68.11 (Glu), 62.43 (Glu), 50.02 (NCH_2_), 30.45 (CH_2_), 22.13 (CH_3_-Ac), 20.96 (CH_3_-Ac), 20.80 (CH_3_-Ac), 20.75 (CH_3_-Ac). HRMS (positive ions, monoisotopic peak): *m*/*z* 1160.1664 [Au_2_L^2^_2_I_2_]^2+^ (calculated for C_74_H_96_Au_2_I_2_N_8_O_36_^2+^ = 1160.1668), 1287.0697 [Au_2_L^2^_2_I_4_]^2+^ (calculated for C_74_H_96_Au_2_I_4_N_8_O_36_^2+^ = 1287.0713), 1266.0660 [Au_2_L^2^_2_I_4_(-CH_2_CO)]^2+^ (calculated for C_72_H_94_Au_2_I_4_N_8_O_35_^2+^ = 1266.0660).

**5**. ^1^H-NMR (300 MHz, CD_3_CN) δ 7.84 (s, 4H, Im), 7.68 (s, 4H, Im), 6.39 (d, *^3^J* = 9.3 Hz, 4H, 1-Glu), 5.80 (t, *^3^J* = 9.6 Hz 4H, 3-Glu), 5.45–5.19 (m, 8H, 2,4-Glu), 4.61–4.42 (m, 4H, NCH_2_), 4.42–4.27 (m, 4H, NCH_2_), 4.27–4.05 (m, 12H, 5,6-Glu), 2.71–2.48 (m, 4H, CH_2_), 2.05 (s, 12H, CH_3_-Ac), 2.01 (s, 12H, CH_3_-Ac), 1.93 (s, 12H, CH_3_-Ac), 1.90 (s, 12H, CH_3_-Ac). ^13^C{^1^H}-NMR (75 MHz, CD_3_CN) δ 171.37 (CO), 171.01 (CO), 170.66 (CO), 170.63 (CO), 154.57 (NCN-Im), 126.14 (Im), 122.99 (Im), 85.68 (1-Glu), 76.16 (Glu), 73.56 (Glu), 72.41 (Glu), 68.10 (Glu), 62.40 (6-Glu), 49.46 (NCH_2_), 31.56 (CH_2_), 21.50 (CH_3_-Ac), 20.80 (CH_3_-Ac), 20.76 (CH_3_-Ac). HRMS (positive ions, monoisotopic peak): *m*/*z* 1033.2615 [Au_2_L^2^_2_]^2+^ (calculated for C_74_H_96_Au_2_N_8_O_36_^2+^ = 1033.2624), 1103.1970 [Au_2_L^2^_2_Cl_4_]^2+^ (calculated for C_74_H_96_Au_2_Cl_4_N_8_O_36_^2+^ = 1103.2001).

### 3.6. Cytotoxicity Assay

To assess the cytotoxicity of the compounds, both immortalized and tumorigenic cells were chosen. Immortalized human keratinocytes (HaCaT, from Innoprot, Derio, Spain), immortalized murine fibroblasts (BALB/c 3T3, from ATCC, Manassas, Vi, USA), human epidermoid carcinoma cells (A431, from ATCC), and BALB/c-3T3 transformed with simian virus 40 (SV40) (SVT2, from ATCC) were cultured in Dulbecco’s modified Eagle’s medium (DMEM) (Sigma-Aldrich, St Louis, MO, USA) supplemented with 10% fetal bovine serum (HyClone, Logan, UT, USA), 2 mM l-glutamine and antibiotics. Cells were grown in a 5% CO_2_ humidified atmosphere at 37 °C and seeded in 96-well plates at a density of 2 × 10^3^ cells per well. Cells were incubated with increasing concentrations of each compound (from 10 to 200 µg·mL^−1^). After 4 h of incubation, cell viability was measured using the MTT (3-(4,5-dimethylthiazol-2-yl)-2,5-diphenyltetrazolium bromide) assay, which measures mitochondrial functionality. Briefly, the MTT reagent was dissolved in DMEM in the absence of phenol red (Sigma-Aldrich) and added to the cells (0.5 mg·mL^-1^ final concentration). Following 4 h of incubation at 37 °C, the culture medium was removed and the resulting formazan salts were dissolved by adding isopropanol containing 0.01 mol·L^−1^ HCl (100 μL per well). Absorbance values were determined at 570 nm using an automatic plate reader (Microbeta Wallac 1420, PerkinElmer, Waltham, MA, USA). 

## 4. Conclusions

In this work, we reported two novel diNHC precursors with one or both the heterocyclic rings functionalized with a carbohydrate moiety. The corresponding neutral complexes of the type Au_2_Br_2_(diNHC) and, with the symmetric ligand, also the dicationic complex [Au_2_(diNHC)_2_](PF_6_)_2_ were synthesized. The antiproliferative properties of these complexes were investigated. Results suggest that the complexes appear rather inert and the introduction of a carbohydrate moiety does not significantly improve their performance. The investigation of the coordinating properties of the new ligands described in this work will be extended to other metal centers in the future.

## Figures and Tables

**Table 1:** IC_50_ values (µM) obtained for **1**, **2**, **3**, and **6** on HaCaT, BALB/c 3T3, A431, and SVT2 cells line after 48 h of incubation.

Cell Line	Complex 1	Complex 2	Complex 3	Complex 6
**HaCaT**	181 ± 8	>144	>85	240 ± 15
**BALB/c 3T3**	148 ± 15	108 ± 17	>85	241 ± 15
**A431**	162 ± 34	137 ± 5	>85	235 ± 16
**SVT2**	139 ± 12	118 ± 6	72 ± 15	207 ± 15

## Supplementary Materials

The following are available online at https://www.mdpi.com/1420-3049/25/17/3850/s1: NMR and ESI-MS spectra of the reported compounds.
