# Supplementary material for: Synthesis and Biological Studies on Dinuclear Gold(I) Complexes with Di-(N-Heterocyclic Carbene) Ligands Functionalized with Carbohydrates
Source: Molecules. 2020 Aug 24;25(17):3850. doi: 10.3390/molecules25173850 (PMC7503629; doi:10.3390/molecules25173850)
Supplement: Supplementary file 1 [file molecules-25-03850-s001.pdf]

Supplementary Information for

Synthesis and Biological Studies on Dinuclear Gold(I) Complexes with Di-(*N*-Heterocyclic Carbene) Ligands Functionalized with Carbohydrates

Federica Tresin [1], Valentina Stoppa [1], Marco Baron [1], Andrea Biffis [1], Alfonso Annunziata,[2] Luigi D'Elia [2], Daria Maria Monti [2], Francesco Ruffo [2], Marco Roverso [1], Paolo Sgarbossa [3], Sara Bogialli [1], Cristina Tubaro [1,*]

[1] Department of Chemical Sciences, University of Padova, via Marzolo 1, 35131 Padova (Italy).
[2] Department of Chemical Sciences, University of Napoli Federico II, Complesso Universitario di Monte S. Angelo, via Cintia 21, 80126 Napoli (Italy).
[3] Department of Industrial Engineering, University of Padova, via Marzolo 9, 35131 Padova (Italy).

* Correspondence: cristina.tubaro@unipd.it; Tel.: +39-049-8275655 (C.T.).

NMR Spectra and ESI-MS spectra for the new compounds

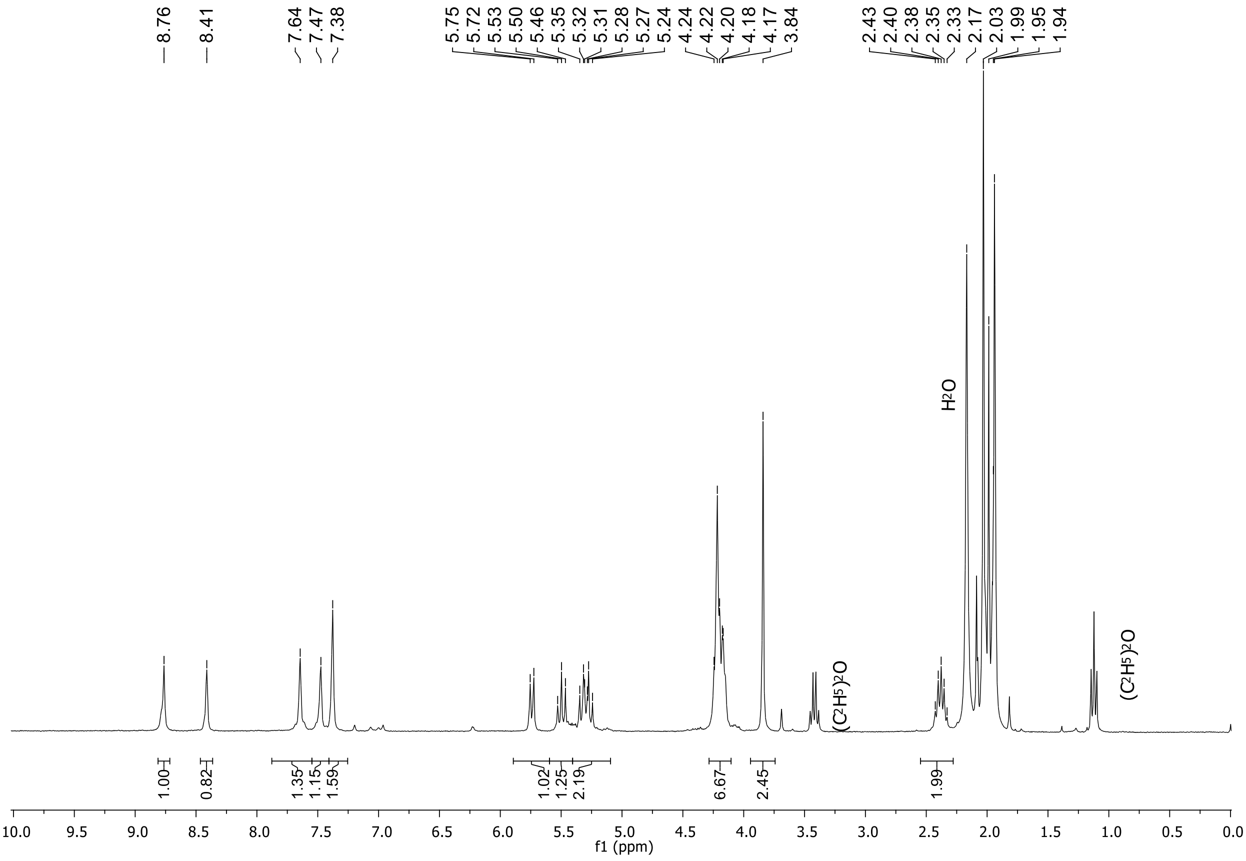

Figure S1: ^{1}H NMR spectra of **L^1·2HPF$_6$** in CD_3CN

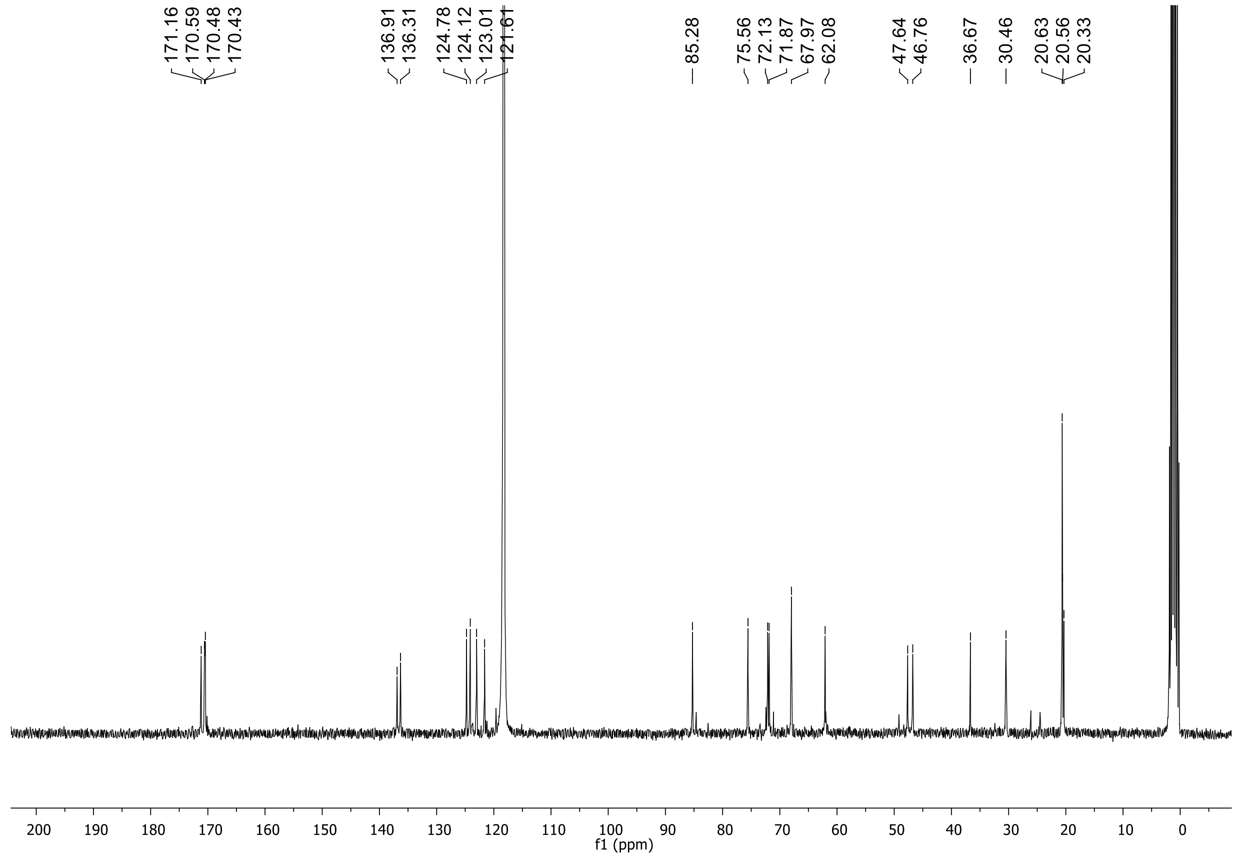

Figure S2: ^{13}C NMR spectra of **L^1·2HPF$_6$** in CD_3CN

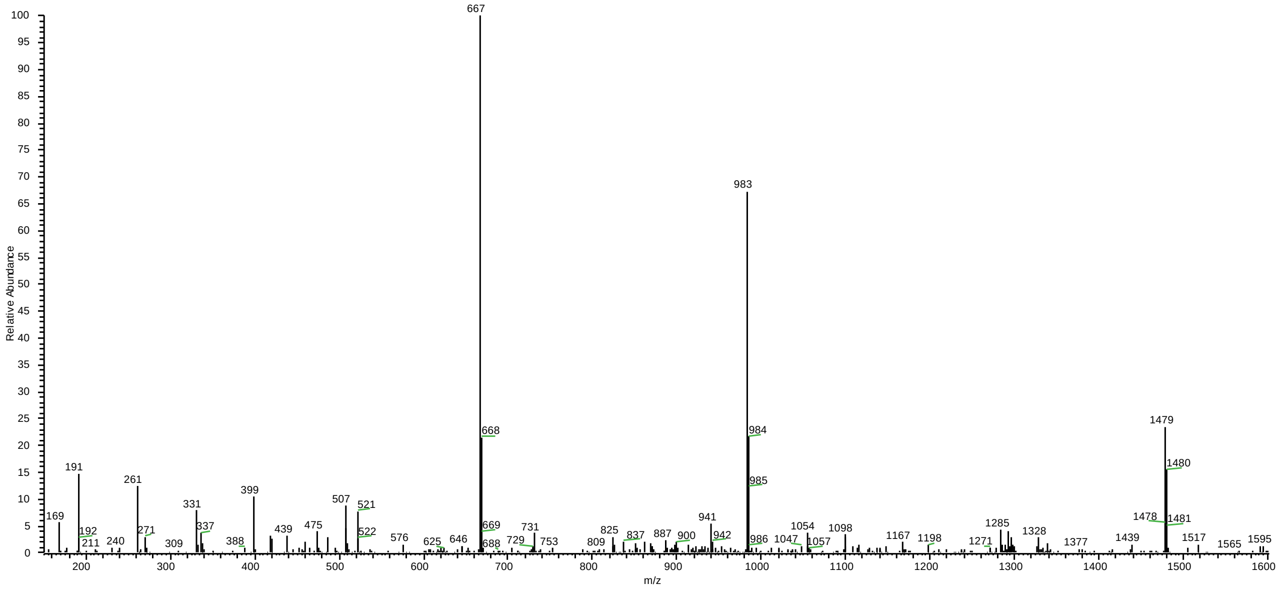

Figure S3: ESI-MS spectra of **L^1·2HPF$_6$**

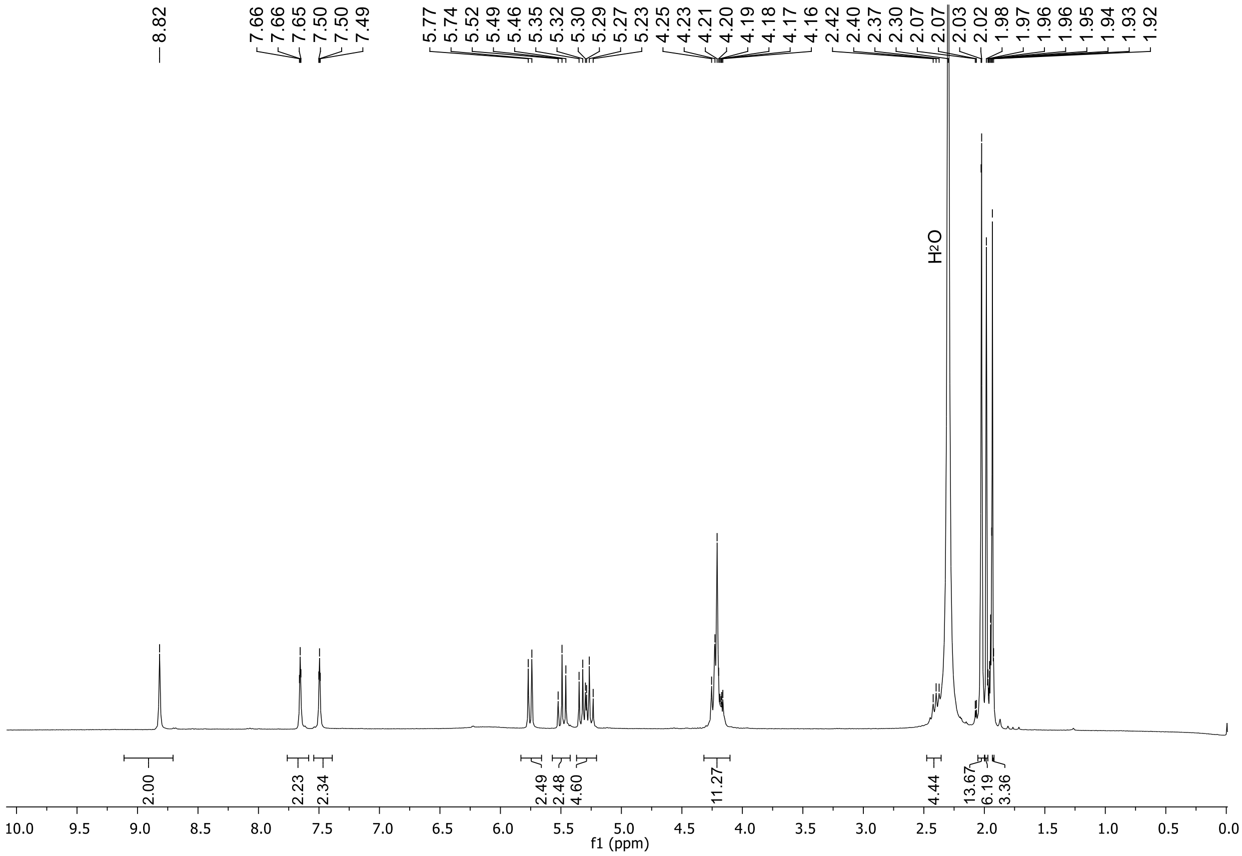

Figure S4: ^{1}H NMR spectra of **L^{2}·2HPF$_{6}$** in CD_3CN

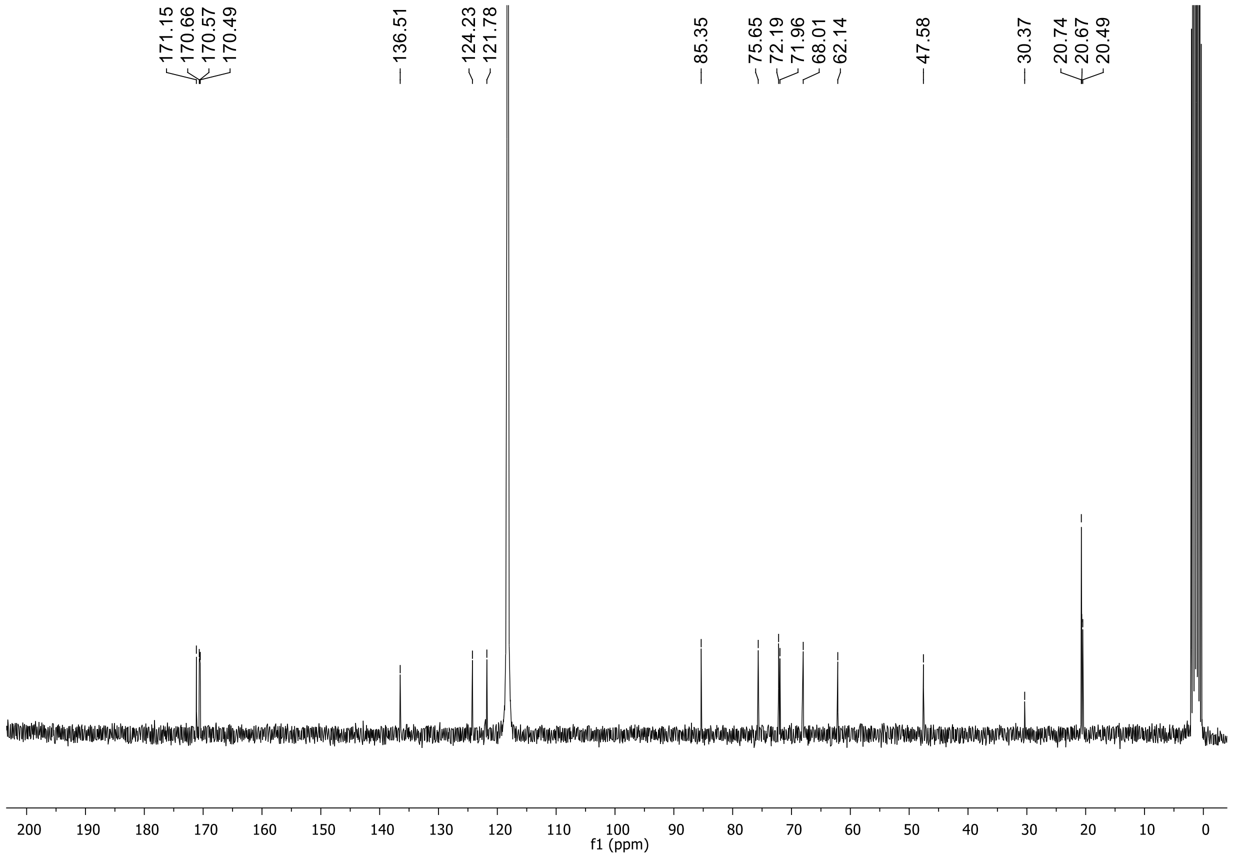

Figure S5: ^{13}C NMR spectra of **L^{2}·2HPF$_{6}$** in CD_3CN

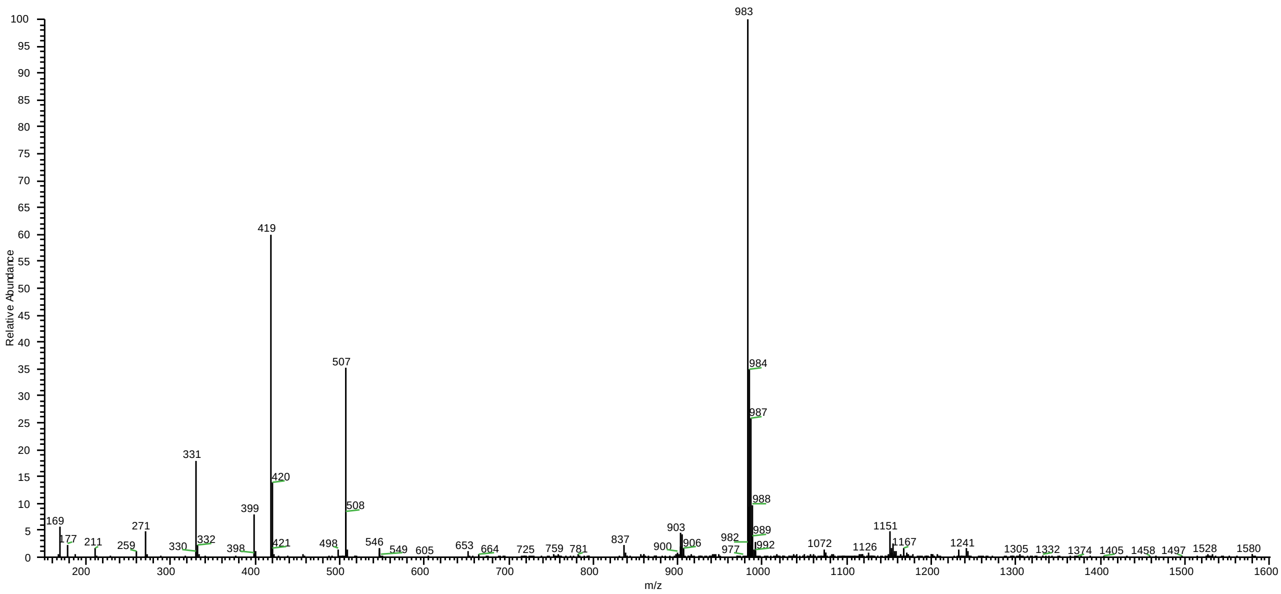

Figure S6: ESI-MS spectra of $\mathbf{L^{2}\cdot 2HPF_{6}}$

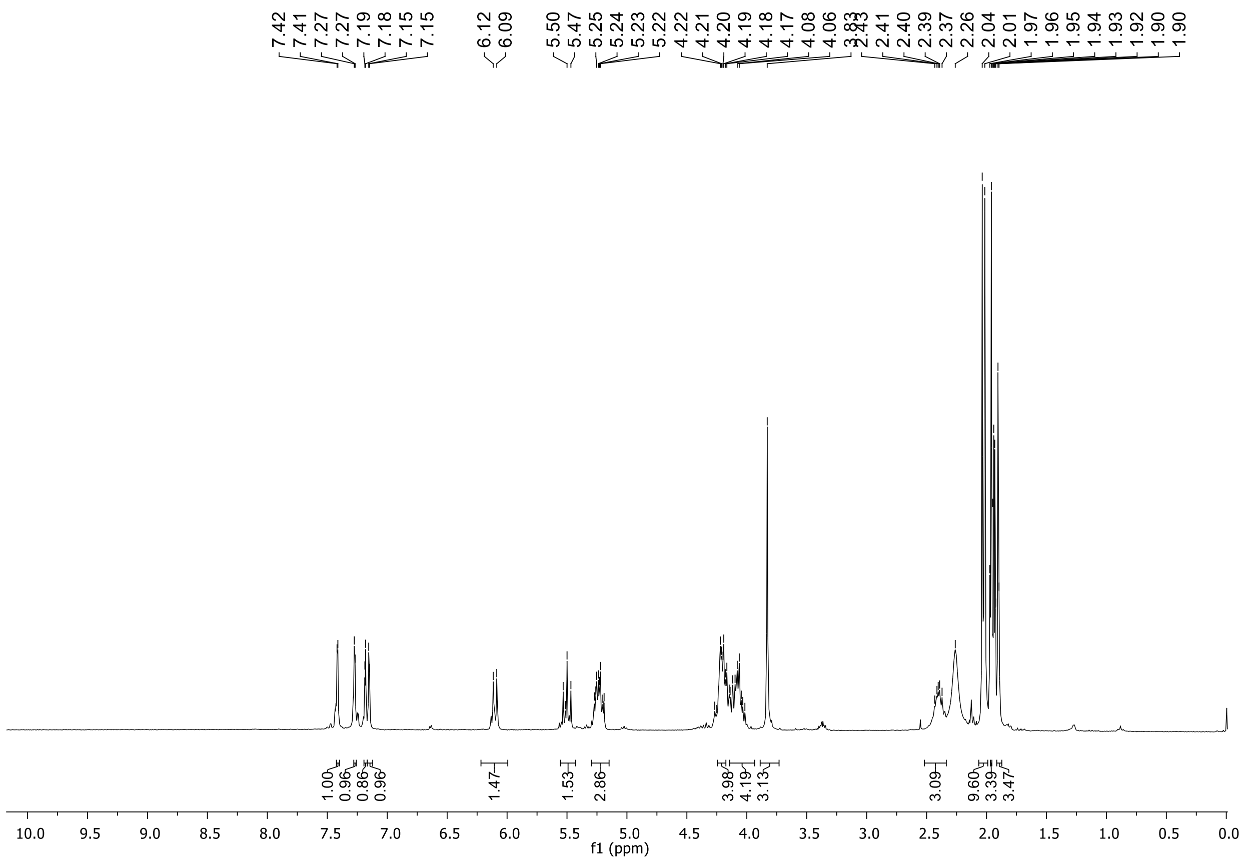

Figure S7: ^{1}H NMR spectra of complex **1** in CD_3CN

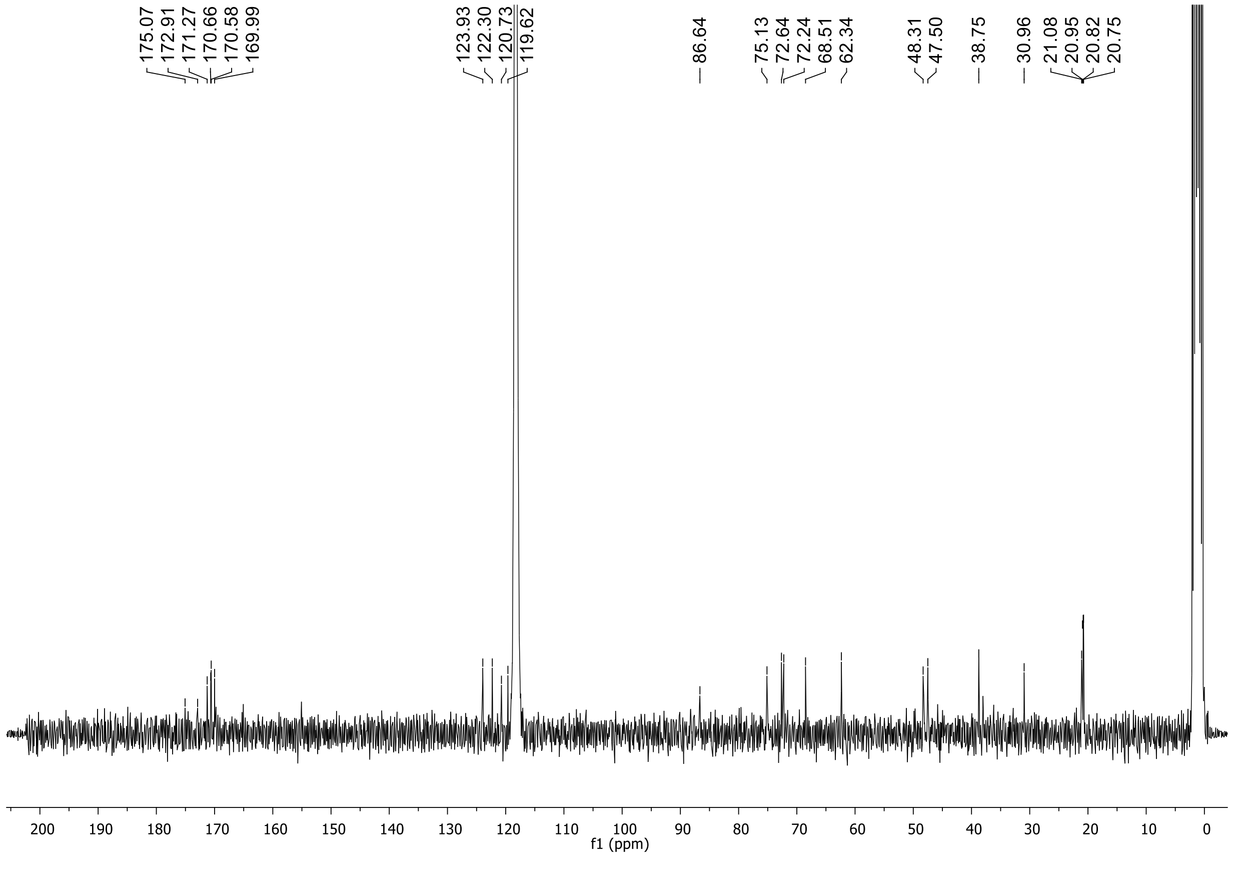

Figure S8: ^{13}C NMR spectra of complex **1** in CD_3CN

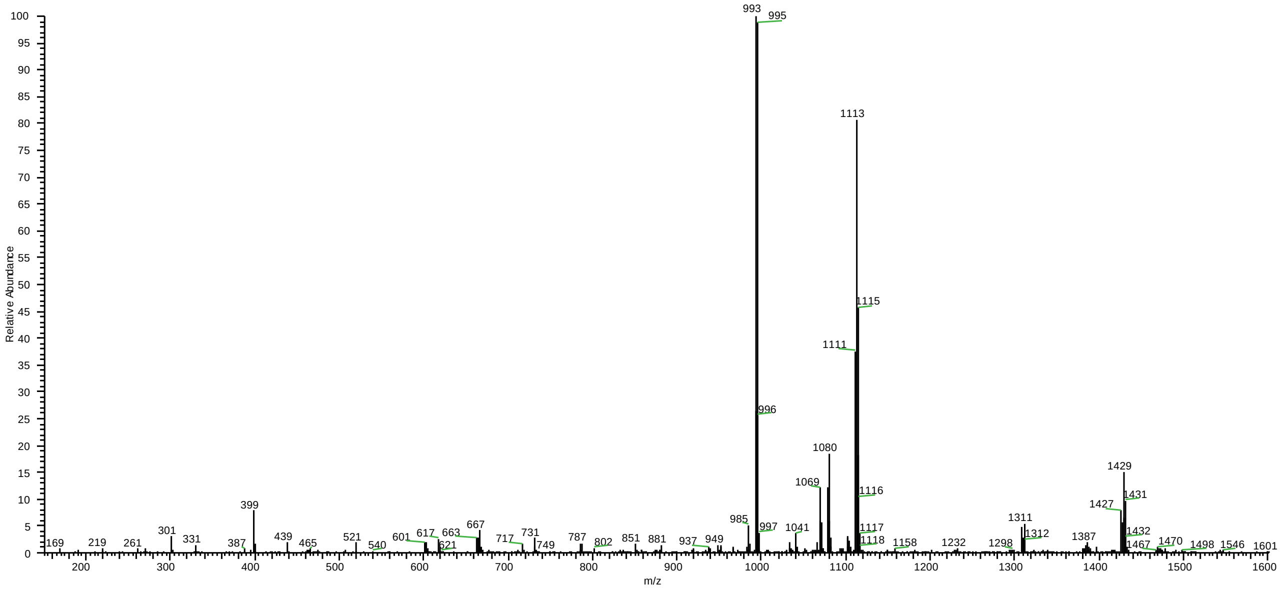

Figure S9: ESI-MS spectra of complex **1**

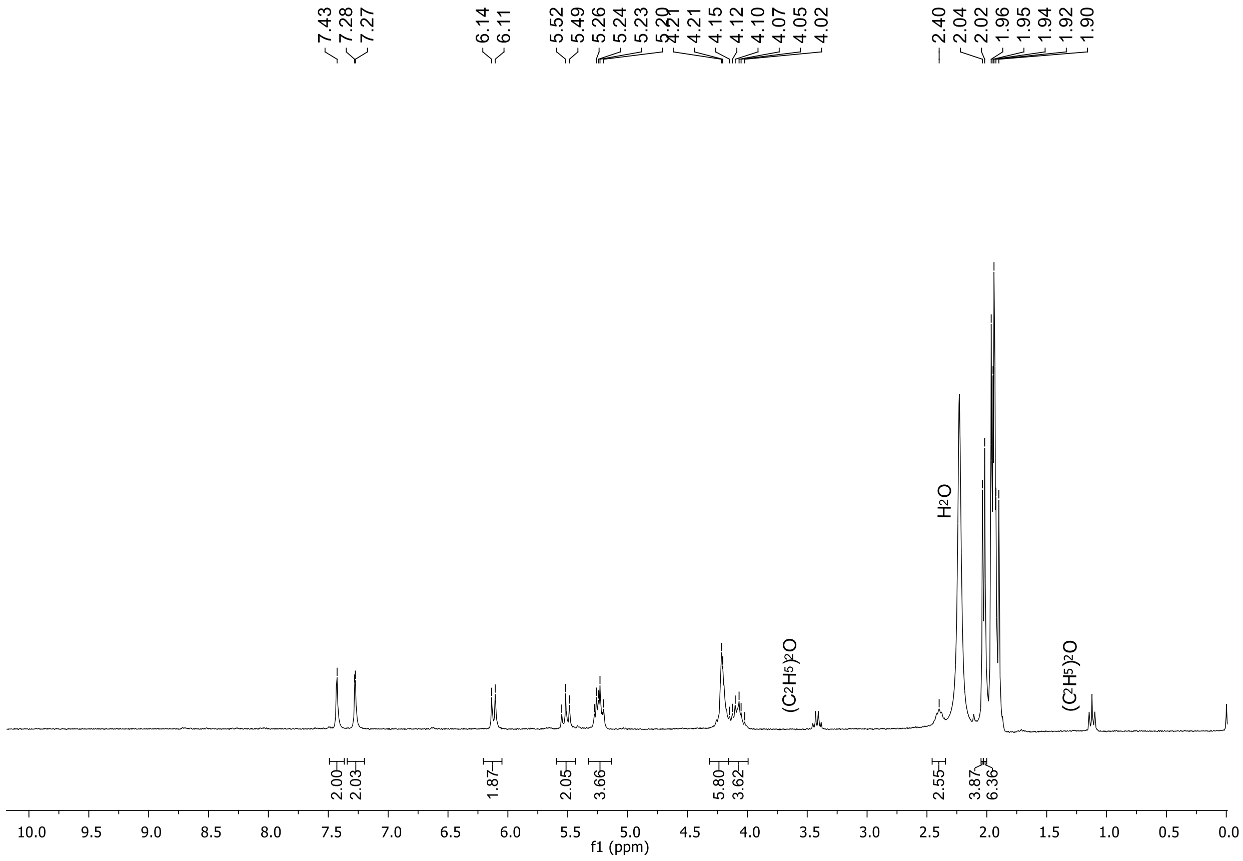

Figure S10: ^{1}H NMR spectra of complex **2** in CD_3CN

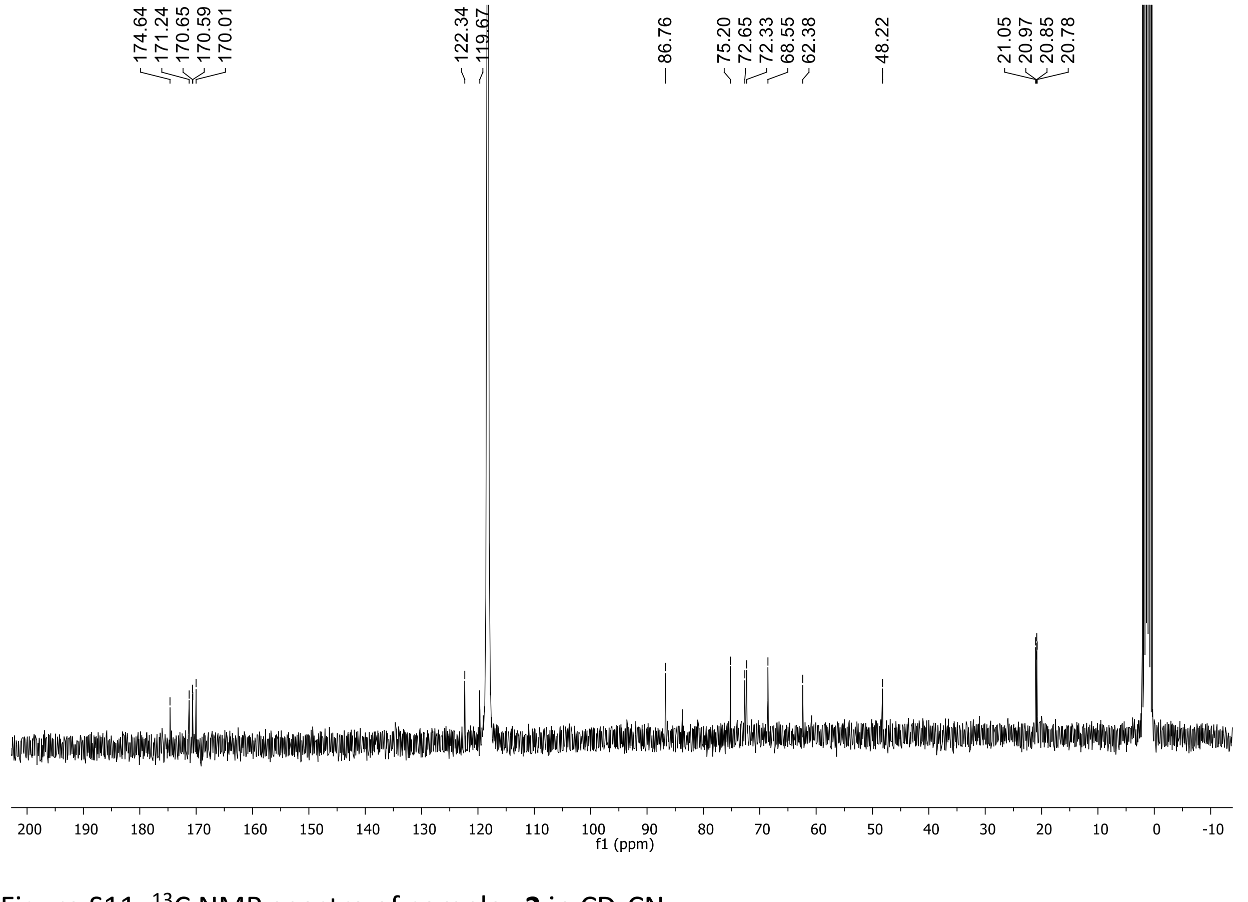

Figure S11: ^{13}C NMR spectra of complex **2** in CD_3CN

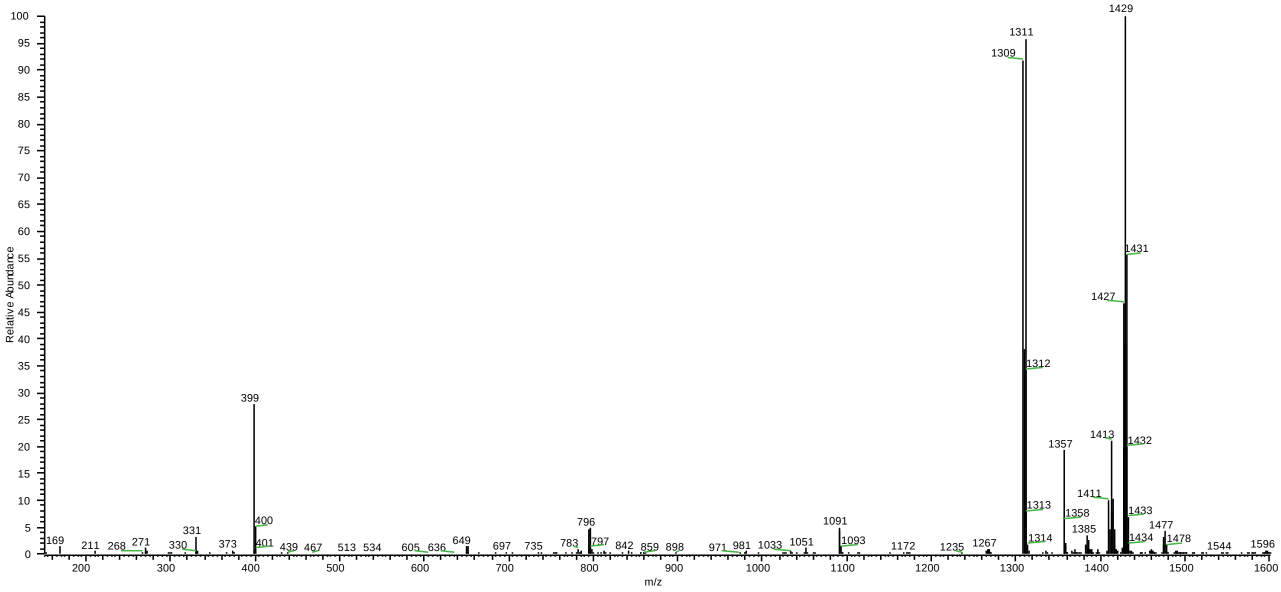

Figure S12: ESI-MS spectra of complex **2**

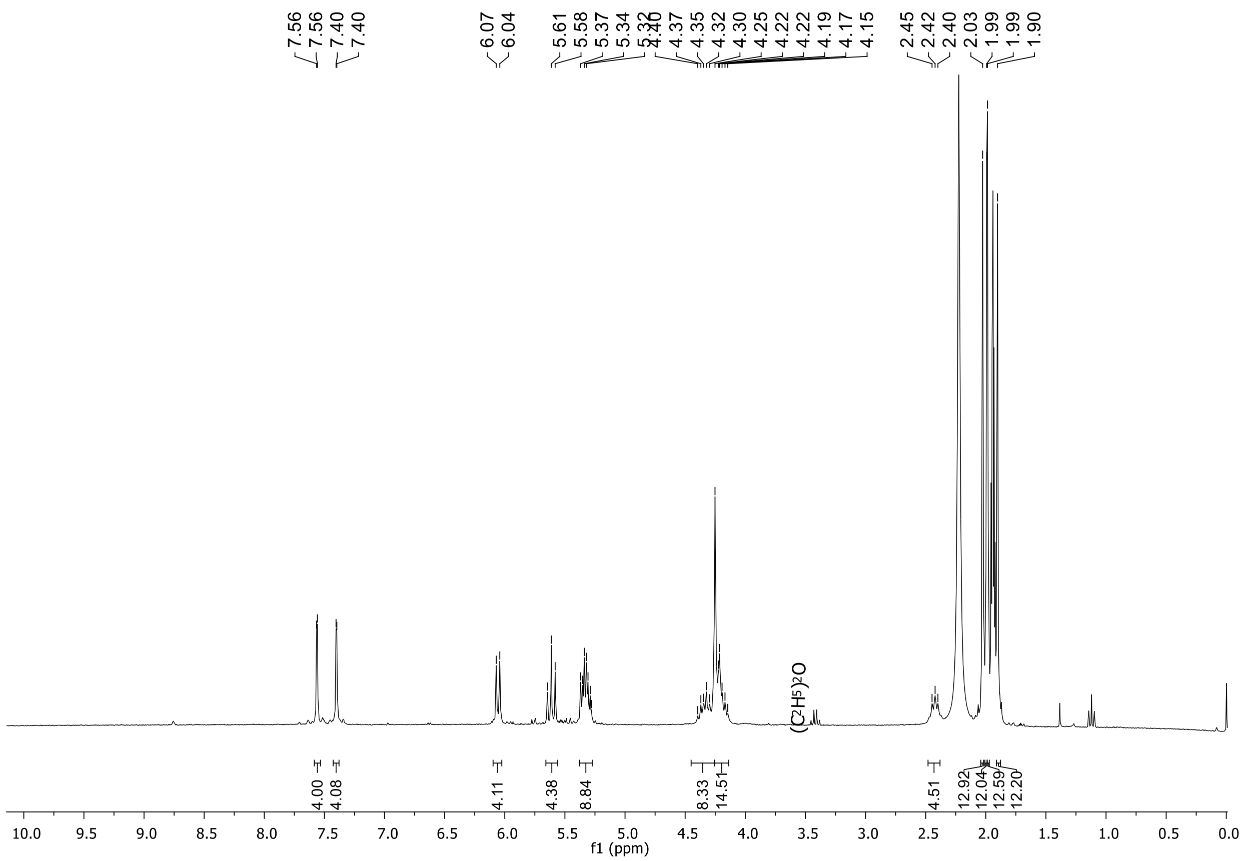

Figure S13: ^{1}H NMR spectra of complex **3** in CD_3CN

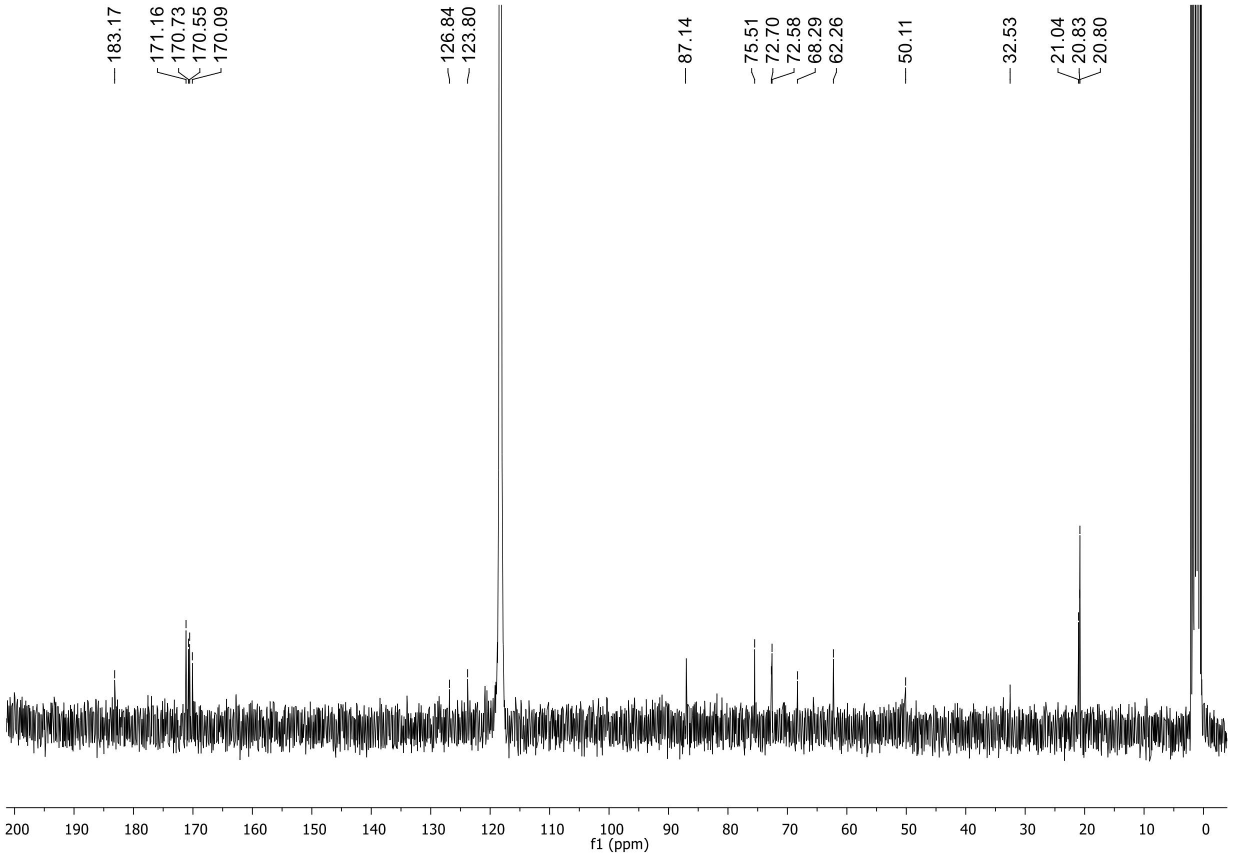

Figure S14: ^{13}C NMR spectra of complex **3** in CD_3CN

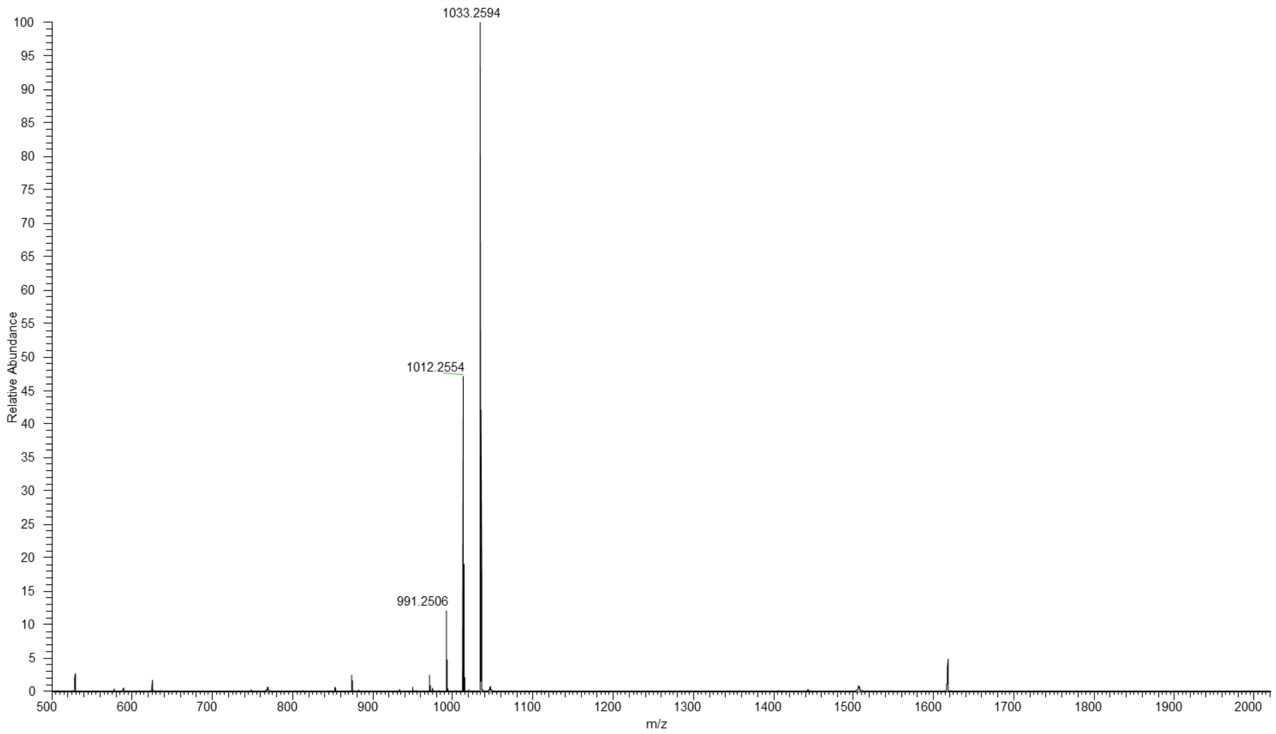

Figure S15: HRMS of complex **3** in CH_3CN

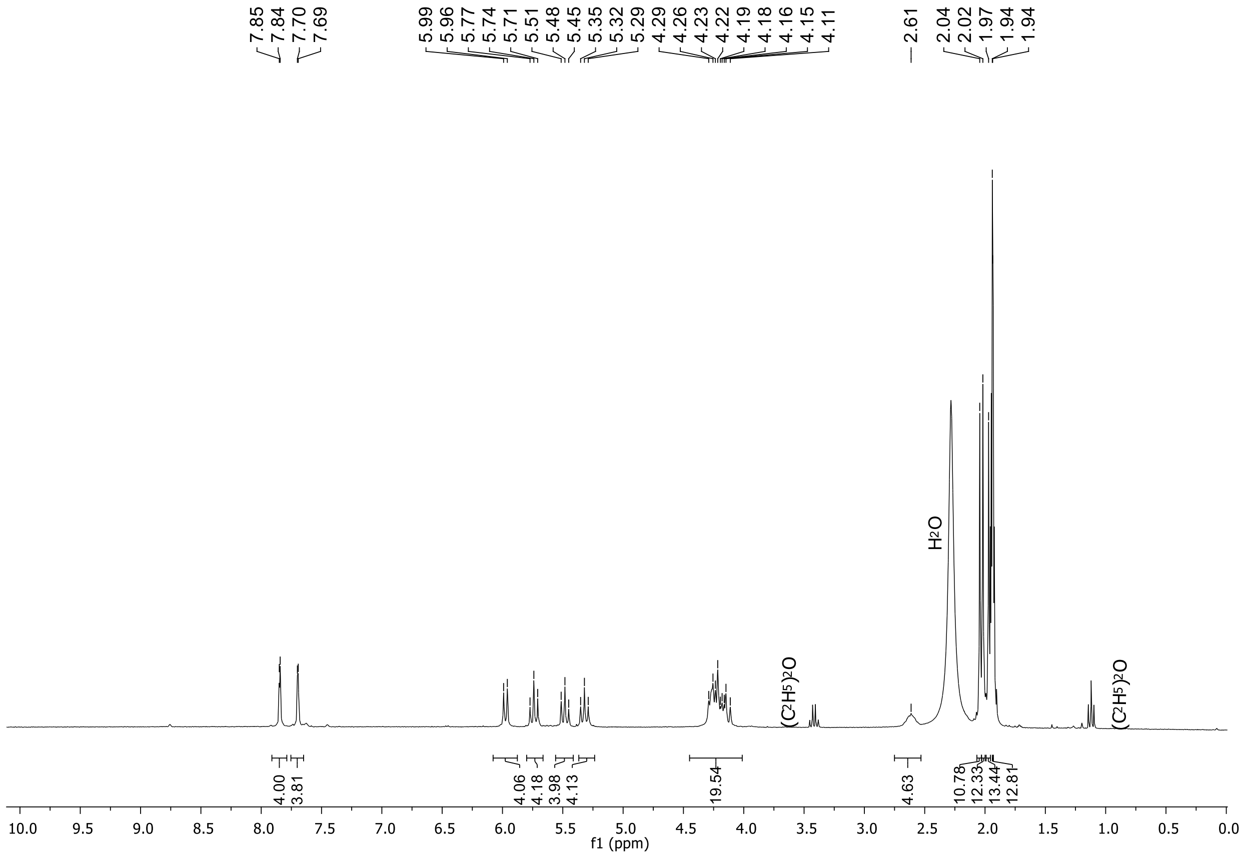

Figure S16: ^{1}H NMR spectra of complex **4** in CD_3CN

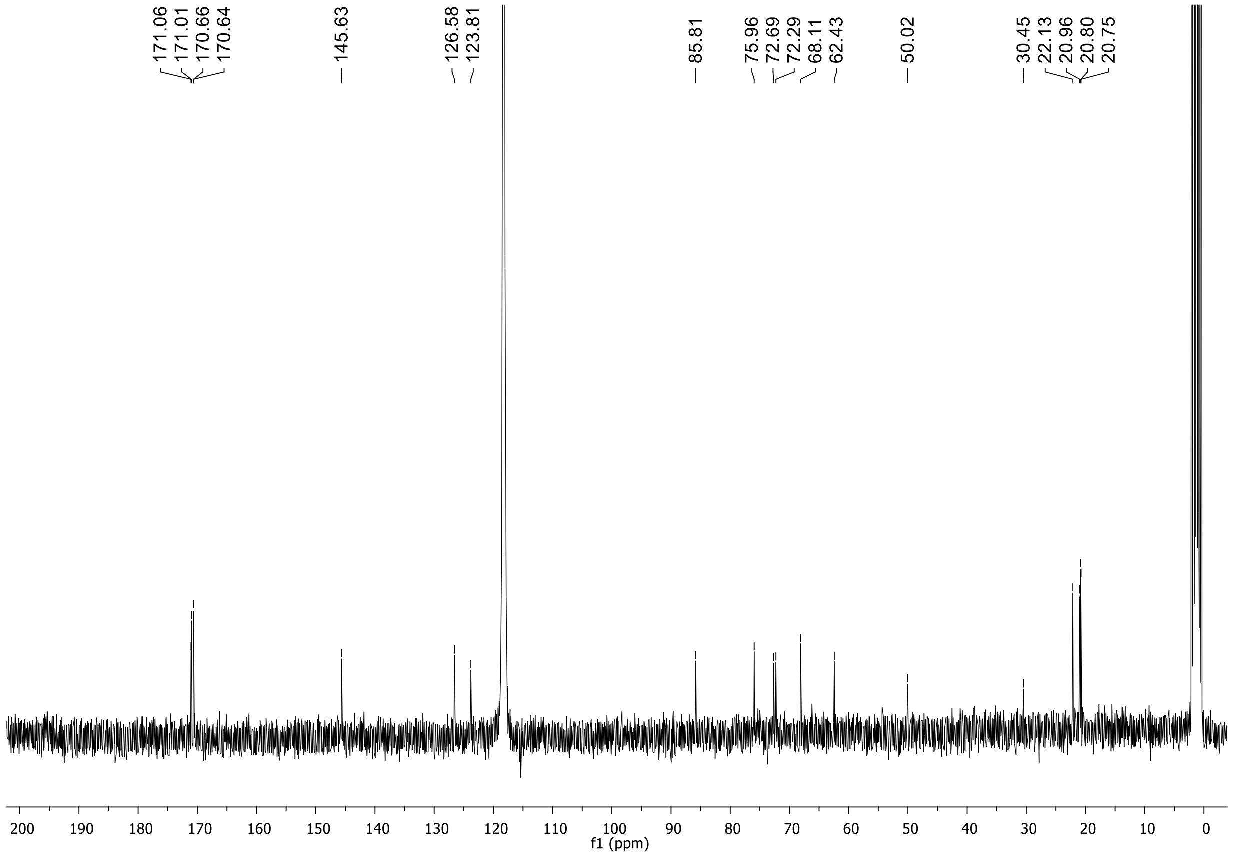

Figure S17: ^{13}C NMR spectra of complex **4** in CD_3CN

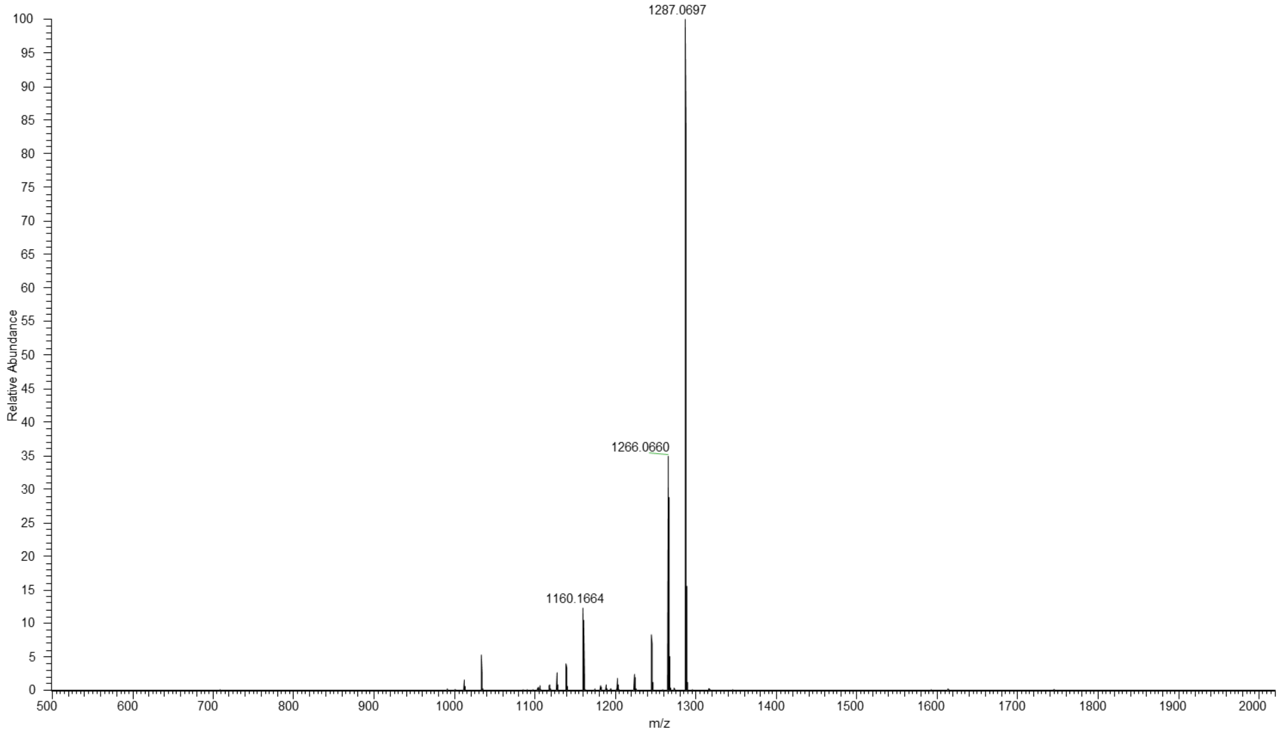

Figure S18: HRMS of complex **4** in CH_3CN

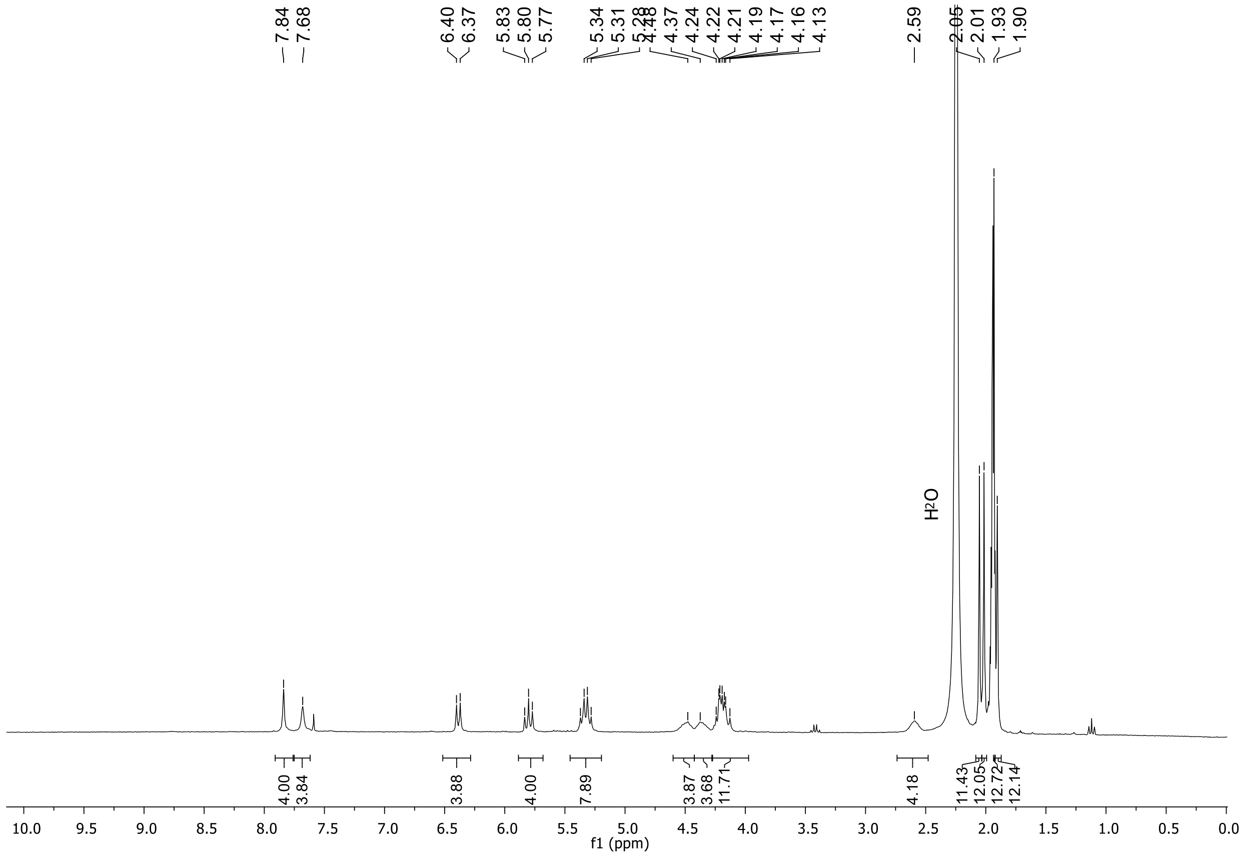

Figure S19: ^{1}H NMR spectra of complex **5** in CD_3CN

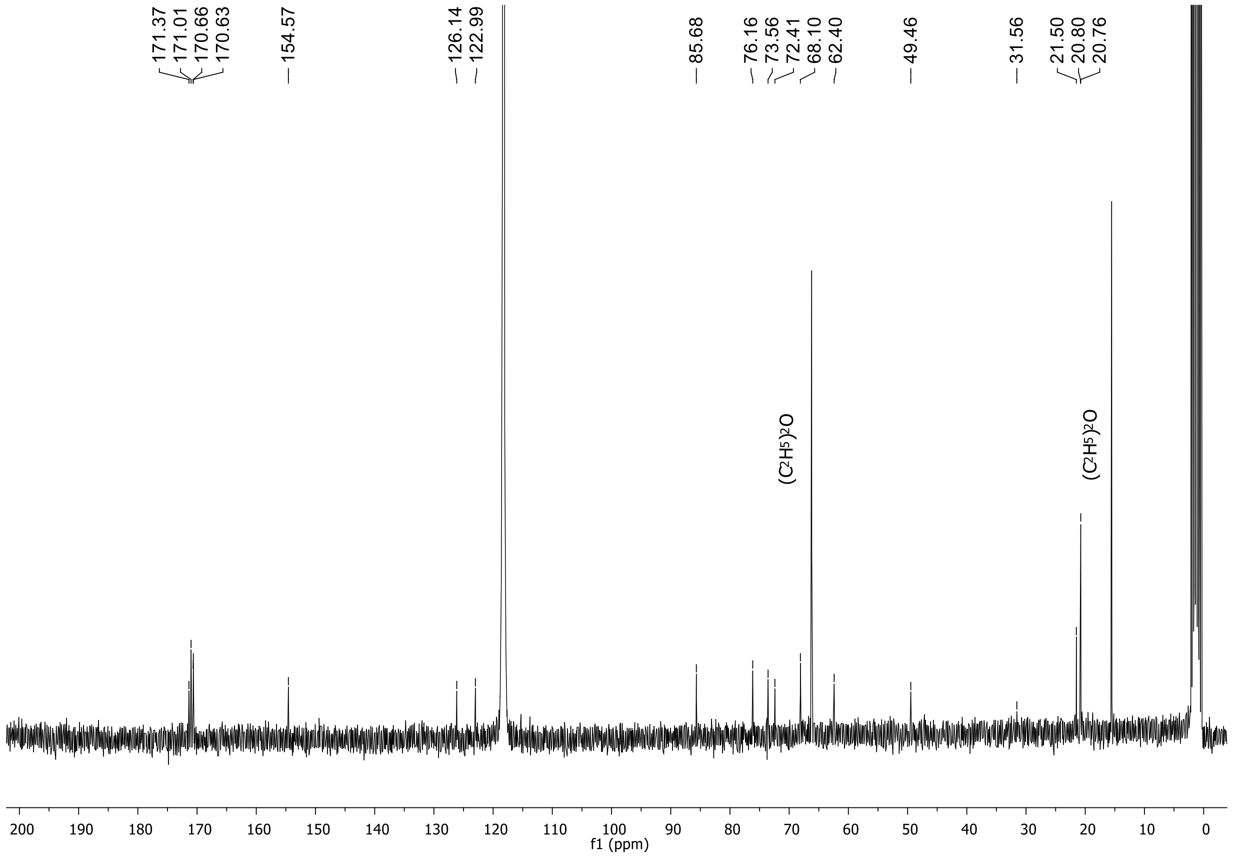

Figure S20: ^{13}C NMR spectra of complex **5** in CD_3CN

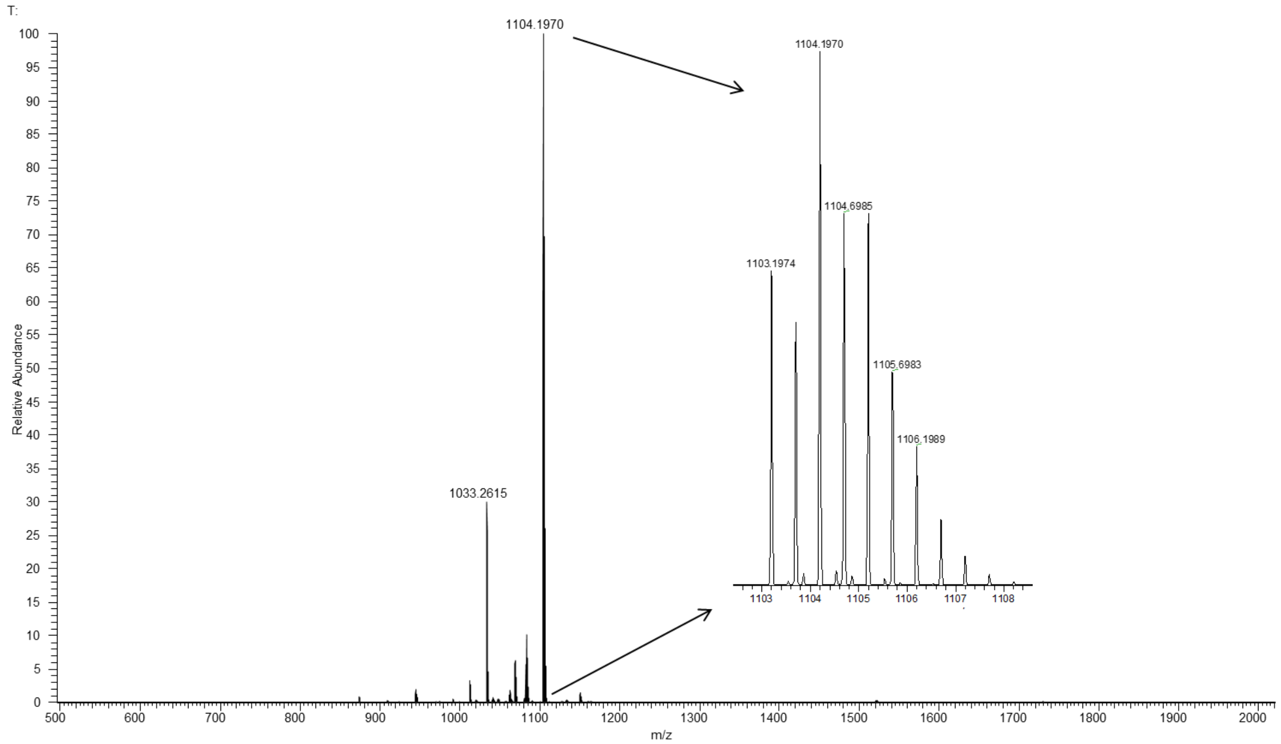

Figure S21: HRMS of complex **5** in CH_3CN
